# Identification of Reprogrammed Myeloid Cell Transcriptomes in NSCLC

**DOI:** 10.1371/journal.pone.0129123

**Published:** 2015-06-05

**Authors:** Anna Durrans, Dingcheng Gao, Ravi Gupta, Kari R. Fischer, Hyejin Choi, Tina El Rayes, Seongho Ryu, Abu Nasar, Cathy F. Spinelli, Weston Andrews, Olivier Elemento, Daniel Nolan, Brendon Stiles, Shahin Rafii, Navneet Narula, Ramana Davuluri, Nasser K. Altorki, Vivek Mittal

**Affiliations:** 1 Department of Cardiothoracic Surgery, Weill Cornell Medical College of Cornell University, 1300 York Avenue, New York, New York 10065, United States of America; 2 Department of Cell and Developmental Biology, Weill Cornell Medical College of Cornell University, 1300 York Avenue, New York, New York 10065, United States of America; 3 Neuberger Berman Lung Cancer Center, Weill Cornell Medical College of Cornell University, 1300 York Avenue, New York, New York 10065, United States of America; 4 Molecular and Cellular Oncogenesis Program, The Wistar Institute, 3601 Spruce St, Philadelphia, PA 19104, United States of America; 5 Weill Cornell Graduate School of Medical Sciences, Weill Cornell Medical College of Cornell University, 1300 York Avenue, New York, New York 10065, United States of America; 6 HHMI, Department of Genetic Medicine, Weill Cornell Medical College of Cornell University, 1300 York Avenue, New York, New York 10065, United States of America; 7 Institute for Computational Biomedicine, Department of Physiology and Biophysics, Weill Cornell Medical College of Cornell University, 1300 York Avenue, New York, New York 10065, United States of America; 8 Department of Pathology, Weill Cornell Medical College of Cornell University, 1300 York Avenue, New York, New York 10065, United States of America; King's College London, UNITED KINGDOM

## Abstract

Lung cancer is the leading cause of cancer related mortality worldwide, with non-small cell lung cancer (NSCLC) as the most prevalent form. Despite advances in treatment options including minimally invasive surgery, CT-guided radiation, novel chemotherapeutic regimens, and targeted therapeutics, prognosis remains dismal. Therefore, further molecular analysis of NSCLC is necessary to identify novel molecular targets that impact prognosis and the design of new-targeted therapies. In recent years, tumor “activated/reprogrammed” stromal cells that promote carcinogenesis have emerged as potential therapeutic targets. However, the contribution of stromal cells to NSCLC is poorly understood. Here, we show increased numbers of bone marrow (BM)-derived hematopoietic cells in the tumor parenchyma of NSCLC patients compared with matched adjacent non-neoplastic lung tissue. By sorting specific cellular fractions from lung cancer patients, we compared the transcriptomes of intratumoral myeloid compartments within the tumor bed with their counterparts within adjacent non-neoplastic tissue from NSCLC patients. The RNA sequencing of specific myeloid compartments (immature monocytic myeloid cells and polymorphonuclear neutrophils) identified differentially regulated genes and mRNA isoforms, which were inconspicuous in whole tumor analysis. Genes encoding secreted factors, including osteopontin (OPN), chemokine (C-C motif) ligand 7 (CCL7) and thrombospondin 1 (TSP1) were identified, which enhanced tumorigenic properties of lung cancer cells indicative of their potential as targets for therapy. This study demonstrates that analysis of homogeneous stromal populations isolated directly from fresh clinical specimens can detect important stromal genes of therapeutic value.

## Introduction

Lung cancer is the leading cause of cancer related mortality worldwide, with an estimated 1.3 million new cases each year [1, 2]. Despite improvements in diagnostics and treatment options [3, 4], 5-year survival rate for lung cancer patients only increased from 7 to 14% within the last 30 years. Thus, further molecular analysis of NSCLC is necessary to identify novel molecular targets that impact prognosis and the design of new, targeted therapies.

A major research focus in NSCLC has been directed to cancer cell intrinsic properties [5], which has led to the discovery of important driver mutations, and the development of targeted therapies such as the receptor tyrosine kinase (RTK) inhibitors gefitinib/erlotinib (EGFR inhibitors) and crizotinib (EML4-ALK inhibitor) [3]. However, these treatments benefit only the small proportion (5–20%) of patients harboring these driver mutations, and acquired resistance to these therapies presents a major impediment to the effective treatment of NSCLC patients with these mutations [6–8].

Emerging studies from solid tumors including breast and prostate are beginning to recognize that carcinogenesis results from concerted interactions between genetically altered tumor epithelial cells and intratumoral stromal cells, resulting in an “activated/reprogrammed” stroma [9]. Consistent with this notion, analysis of enriched stromal compartments derived from human breast cancer revealed gene expression changes associated with cancer progression [10]. Notably, BM-derived hematopoietic cells contribute significantly to the tumor stroma, and are “educated/reprogrammed” by the paracrine activity of tumor epithelial cells to acquire an “activated” protumorigenic phenotype [11]. Examples of tumor-activated stromal cells include macrophages (activated M2 phenotype) [12], neutrophils (N1 to N2 conversion) [13], lymphocytes [14], fibroblasts (cancer activated fibroblasts, CAFs) [15], and endothelial cells [16]. Studies from mouse models have shown that reprogrammed stromal cells promote tumor growth by regulating key cancer hallmarks such as angiogenesis, proliferation, migration, and invasion [11, 17, 18] leading to the inclusion of the tumor microenvironment as an emerging hallmark of cancer [19]. Furthermore, recent studies have demonstrated that stromal cells mediate innate resistance to therapies in many cancers [20–22]. Importantly, administration of chemotherapy in combination with a macrophage antagonist (CSFR1 blockade) conferred synergy in breast cancer treatment [23]. These studies, together with the clinical success of the antiangiogenic agent bevacizumab, a humanized monoclonal anti-VEGF antibody, provide compelling rationale for targeting the tumor microenvironment. Intratumoral stromal cells have thus emerged as attractive targets for anti-cancer therapy [11, 24].

Little is known, however, about the contribution and pathophysiological role of stromal cells in NSCLC. A few clinical studies have shown that activated stromal elements may determine patient prognosis and may play a role in mediating resistance to targeted therapies. For example, in patients with stage I NSCLC the presence of CAFs is a poor prognostic indicator typically associated with nodal metastases and a higher risk of recurrence [25]. Interestingly, a specific, eleven-gene expression signature in CAFs stratified NSCLC patients into low and high-risk groups, and was associated with recurrence free survival [26]. In another study, the tumor-stroma cross talk was implicated in mediating resistance to EGFR-TKIs [27].

In NSCLC, genetic features of tumor epithelial cells continue to be used for both prognosis and the development of targeted therapies; however, another important question is how to exploit the reprogrammed intratumoral stromal compartments, which play pivotal roles in carcinogenesis. New approaches are required to identify and isolate individual cellular stromal components from the heterogeneous tumor, so that their protumorigenic characteristics can be elucidated and targeted for prognostic and therapeutic purposes. Recognizing that the contribution of BM-derived cells to NSCLC progression has not been widely studied, we set out to study specific individual populations of BM-derived myeloid cells within the tumor and adjacent non-neoplastic tissue from fresh clinical material obtained from NSCLC patients, and assessed their contribution and function in NSCLC.

## Methods

### Human samples

Human tumor and adjacent non-neoplastic lung samples were obtained from the Cardiothoracic Surgery Department, Weill Cornell Medical College (New York). Specimens were collected after obtaining written informed consent prior to undergoing any study-specific procedures in accordance with the Declaration of Helsinki. Patient’s identity of pathological specimens remained anonymous in the context of this study. Patient sample collection was approved by the Institutional Review Board of Weill Cornell Medical College, Thoracic Surgery Biobank Protocol Number 1008011221.

### Mice and cell lines

All animal work was conducted in accordance with a protocol approved by the Institutional Animal Care and Use Committee at Weill Cornell Medical College. Wild type C57BL/6J and p53^f/f^ (B6.129P2-*Trp53* ^*tm1Brn*^/J) mice were obtained from The Jackson Laboratory (Bar Harbor, Maine), and K-ras (B6.129S4-Kras^tm4Tyj^/J (LSL Kras-G12D)) mice from The National Cancer Institute (Bethesda, Maryland). The p53^f/f^ and K-ras mice were then bred together for 3 generations to get Kras-Het; p53f/f mice.

The HKP-1M cell line was established by culturing cells derived from lung adenocarcinomas from mice which constitutively express oncogenic K-ras and have loss of function of p53. Cells were cultured in DMEM with 10% fetal bovine serum. The human non-small cell lung cancer cell line (H1650) was obtained from ATCC and cultured in RPMI with 10% fetal bovine serum.

### Immunofluorescence and microscopy

For immunofluorescence staining, tissues were fixed in 3.7% formalin overnight, cryoprotected in 30% sucrose/PBS overnight, and then cryoembedded in a 1:1 solution of 30% sucrose/PBS:Tissue-Tek O.C.T. embedding compound (Electron Microscopy Sciences). To obtain lung tissue from animals, perfusion was performed by injecting 5 ml of cold PBS through the right ventricle of the heart before embedding.

Sections (12 μm) were stained following a standard protocol with primary antibodies against EpCAM (anti-human clone 9C4, BioLegend), CD45 (anti-human clone HI30, BioLegend), CD11b (anti-human clone ICRF44, eBioscience), OPN (anti-human clone EPR3688, Epitomics), CCL7 (anti-human rabbit polyclonal, Abcam), and TSP1 (anti-human clone Ab-11, ThermoScientific).

Unconjugated primary antibodies were directly conjugated to various Alexa Fluor dyes using antibody-labeling kits (Invitrogen) performed as per manufacturer’s instructions and purified over BioSpin P30 Gel (Biorad). Fluorescence images were obtained using a computerized Zeiss fluorescence microscope (Axiovert 200M), fitted with an apotome and a HRM camera. Images were analyzed using Axiovision 4.6 software (Carl Zeiss Inc.).

For H&E staining, lung sections (5 μm) were stained with hematoxylin and eosin dyes following standard protocols. Images were obtained with an Olympus BX51 microscope coupled with Qcapture software (Olympus).

To obtain single cell suspensions, fresh clinical tissues were minced and then digested in a collagenase/dispase/DNase mix (Roche) for 30 min at 37°C. Cells were washed, strained through a 70-μm strainer (BD Bioscience) and resuspended in FACS staining buffer (PBS, 2mM EDTA, 1%BSA). Red blood cells were eliminated by incubation in lysis buffer (5 PRIME) for 10 min at RT. Cell suspensions were pre-blocked with 1% FBS and then incubated with the following primary antibodies from BioLegend: CD45 (anti-human clone HI30), EpCAM (anti-human clone 9C4), CD33 (anti-human clone WM-53), CD11b (anti-human clone ICRF44, anti-mouse clone M1/70), CXCR2 (anti-human clone 5E8), Ly6G (anti-mouse clone 1A8), Ly6C (anti-mouse clone HK1.4), CD4 (anti-mouse clone GK1.5), CD8a (anti-mouse clone 53–6.7), and B220 (anti-mouse clone RA3-6B2). SYTOX Blue (Invitrogen) was added in each cell staining tube to facilitate the elimination of dead cells in flow cytometry analysis. Single color stainings were freshly set up with according antibodies and CompBeads (BD Bioscience) in each experiment for proper calibration of compensations.

Labeled cell populations were measured by LSRII flow cytometer coupled with FACS Diva software (BD Bioscience). Flow cytometry analysis was performed using a variety of controls including isotype antibodies, FMO samples [28], and unstained samples for determining appropriate gates, voltages, and compensations required in multivariate flow cytometry. For sorting, targeted cell populations were gated within FACS Diva software and sorted by Aria II sorter (BD Bioscience).

### RNA isolation, quality assessment and RNA-seq library preparation

Total RNA from flow cytometry sorted cells was extracted using the PicoPure RNA extraction kit (Arcturus) following the manufacturer´s protocol. RNA concentration was measured using a nanodrop (Thermo Fisher Scientific), and RNA integrity determined using the Agilent 2100 Bioanalyzer (Agilent Technologies). cDNA libraries were prepared using 15–35 ng RNA starting material (RIN values >6.0), using the TruSeq RNA Sample Preparation Kit (Illumina) according to the manufacturer’s instructions. Libraries were quantified using a Qubit fluorometer (Invitrogen) and the size and purity checked on an Agilent 2100 Bioanalyzer (Agilent Technologies). 19 samples from six patients were prepared for sequencing using the HiSeq 2000 (illumina) (S1 Table).

### RNA-seq data analysis

RNA-seq data analysis was performed according to our published analysis pipeline [29, 30]. Differential gene expression analysis was performed using the DEseq method [31] which is based on a negative binomial distribution model. Approximately 80% of the reads aligned to known and novel mRNAs in each case, confirming high quality mRNA sequencing data. Uniquely mapped reads were used to identify differentially expressed genes (p ≤ 0.05). Importantly, correlation analysis of these genes showed discrete clustering of stromal cells from tumors and adjacent lungs with minimal patient-to-patient variability, suggesting that RNA-seq data from this patient cohort was sufficiently robust to identify differentially regulated genes. Next, genes differentially regulated in the intratumoral stromal subsets and epithelial cells, compared with the same subsets from adjacent lung tissue, were identified using standard statistical methods with a false discovery ratio (FDR) <5%. Ingenuity pathway analysis of differentially regulated genes from stromal compartments showed that a major proportion of genes were involved in cancer-specific pathways (data not shown).

An in-house algorithm was used to select genes from the RNA-seq gene list according to the following criteria; adjusted p value<0.05, fold change >2.0, potential for paracrine function as determined by GO annotation as secreted, extracellular space, or membrane (except membranes of organelles including golgi and endoplasmic reticulum). Of these, genes with functions in key tumorigenic pathways including angiogenesis, ECM breakdown, cell migration, proliferation, invasion, cytokine function, chemokine function, and chemotactic function were selected.

In addition, a systematic gene set enrichment analysis (GSEA) was carried out using the tumor vs. adjacent comparison to rank genes in each population. Specifically we used negative log10 DEseq p-values multiplied by the sign of the log2 fold-changes to rank genes. We applied GSEA to the c5 and c7 gene sets from MSigDB and used FDR = 5% as threshold.

### Quantitative RT-PCR analysis

RNA (50 ng, RIN values >6.0) from individual flow-sorted cell populations was pooled from 3–5 samples and converted to cDNA using the RT^2^ First Strand Kit (SABiosciences). QRT-PCR was performed with RT^2^ SYBR Green Master mix and customized 384 well arrays of 86 specific primers, as well as two housekeeping genes (HPRT1, GAPDH), a control for DNA contamination, a reverse transcription control, and a positive PCR control. The positive PCR control (PPC, SABiosciences) tested the efficiency of the polymerase chain reaction using a pre-dispensed artificial DNA sequence and the primer set that detects it. Any impurities that affect the positive control PCR amplification also affect amplification of the gene-specific products of interest. The custom PCR array was designed with RT^2^ primers (SA Biosciences) which have an average amplification efficiency of 99% with 95% CI from 90–110% and are optimized with the reagents used by SA Biosciences. The array included HPRT1 and GAPDH primers as internal controls. The geometric mean of both genes was used to normalize cDNA input. PCR data was analyzed by using established and validated online tools from www.Qiagen.com.

Each sample was duplicated to control pipetting error. A PCR protocol of initial denaturing at 95°C for 10 min, 40 cycles of 95°C for 15 sec, 60°C for 1 min, and 72°C for 30 sec, followed by final extension at 72°C for 5 min and melt curve analysis was applied on an ABI 7900HT Fast Real-Time PCR System (Applied Biosystems) coupled with SDS 2.0 software. The relative abundance of each transcript compared with the control was calculated utilizing the delta-Ct method.

### Orthotopic model of lung cancer in mice and bioluminescent imaging

To determine the functional significance of myeloid cells in tumor growth we used the *K-ras* ^G12D^ *p53* ^flox/flox^ mouse model of NSCLC [32]. Tumors were excised from these animals and used for deriving cell lines for generating orthotopic lung adenocarcinoma in mice. Murine, myeloid subsets are defined as CD11b^+^Gr1^+^ cells, which are comprised of two major subpopulations defined as CD11b^+^Ly6C^high^ (IMMCs) and CD11b^+^Ly6G^high^ (neutrophils) [18, 33, 34]. To assess the effects of depleting specific myeloid cell populations on NSCLC progression, we used a Ly6G-specific monoclonal antibody which has previously been used for depleting neutrophils in mice [35]. We did not deplete Ly6C^high^ cells, as robust reagents for specifically depleting these cells are unavailable.

8-week old C57BL/6 mice were injected *via* tail vein with 1.5 X 10^5^ luciferase-labeled HKP-1M cells. At D7 treatment was begun with retro-orbital administration of anti-Ly6G antibody or IgG control (2.5 mg/kg, purified rat anti-mouse Ly6G, and rat IgG2a κ isotype control, Becton Dickinson), which was repeated every three days until sacrifice.

To determine the lung tumor burden *in vivo*, mice were anaesthetized and injected retro-orbitally with 75mg/kg of D-luciferin. Lung growth was monitored every 3 days using bioluminescence imaging performed with mice in a supine position by using the Xenogen IVIS system coupled to Living Image acquisition and analysis software (Xenogen). For BLI plots, photon flux was calculated for each mouse by using the same rectangle region of interest encompassing the thorax of the mouse.

### Micro-CT imaging and tumor volume measurements

Mice were anesthetized with a continuous flow of 2–4% isoflurane/oxygen mixture (2.5 L/min), and imaged two at a time using the Siemens Inveon micro PET/CT scanner (Siemens Medical Solutions USA, Inc). The micro-CT image acquisition consisted of 501 projections collected in 203 degree of scan arc. The X-ray exposure time was 1 sec per projection without respiratory-gating. The X-ray tube settings were 30 kVp and 500 μA. The resulting raw data were reconstructed to a final image matrix of 1024 x 1024 x 480 slices at 98-μm voxel size. The reconstructed voxel values were scaled into Hounsfield units. The reconstructed images were viewed and analyzed using the Inveon Research Workspace software (Siemens Medical Solutions USA, Inc).

The image analysis method was based on defining the total lung space, excluding the heart, using manual segmentation. Vascular structures around the heart were manually included in the total lung space. The radiodensity of the combined functional lung, tumor, and vasculature was recorded as Hounsfield units (HU) where -1000 is air, -700 is lung, and +100 to +300 is soft tissue. Because tumor tissue and vascular tissue have similar grayscale values, they could not be separated during segmentation, however non-tumor vascular volume should remain relatively constant between the control and treated groups, and therefore this method provides a relative measure of tumor burden. All data are presented as mean ± SEM.

### Cell proliferation, migration and invasion assays

Human lung cancer cells (H1650) (2×10^5^) were cultured in 6-well plate with or without OPN, CCL7, or TSP1 at a concentration of 100ng/mL for 1 day. For the cell proliferation assay, EdU (10nM) was administered to culture medium to label proliferating cells for 30 min. Cells were harvested, washed once with PBS, and fixed with 4% paraformaldehyde for 15 min at RT. Fixed cells were permeabilized and stained for EdU incorporation and DNA content using the Click-iT EdU Cell Proliferation Assay kit (Invitrogen Inc) according to the standard protocol. Flow analysis of cell phases was performed using an LSRII coupled with Diva software (BD Bioscience).

For the cell migration assay, H1650 cells (2×10^5^) were seeded in 6-well plate (Corning Inc) with or without OPN, CCL7, or TSP1 at different concentrations for 24 hours. A cell migration video was generated by obtaining images every 5 min for 3 hours under a computerized Zeiss microscope (Axio Observer) equipped with a culture chamber. Cell movement was tracked and analyzed using ImageJ and Manual Tracking Plugin software (NIH).

For the cell invasion assay, H1650 (2×10^4^) cells were seeded in 24-well BD BioCoat Matrigel invasion chambers (BD Bioscience) in serum free medium with or without OPN, CCL7, or TSP1 for 20 hours. The lower chambers contained 5% serum for attracting tumor cell invasion for 8 hours. The chambers were fixed and stained with hematoxylin to visualize the invading cells.

### Statistical Analysis

Results are expressed as mean ± SEM, except where indicated otherwise. Analyses of different treatment groups were performed using the Mann-Whitney *t*-test using the GraphPad Prism statistical program. P values < 0.05 were considered significant.

## Results

### Increased numbers of bone marrow hematopoietic cells infiltrate lung adenocarcinoma

To evaluate the cellular landscape of tumor-activated BM stromal cells in human NSCLC we used stringent criteria for sample selection, preparation, and analysis. For sample selection the criteria used were samples from patients who had; 1) surgical resection without preoperative therapy, 2) stage I/II/III disease, 3) adenocarcinoma cell type, and were, 4) never or former smokers (to exclude confounding smoking-induced acute inflammatory reactions in active smokers).

**Fig 1:**
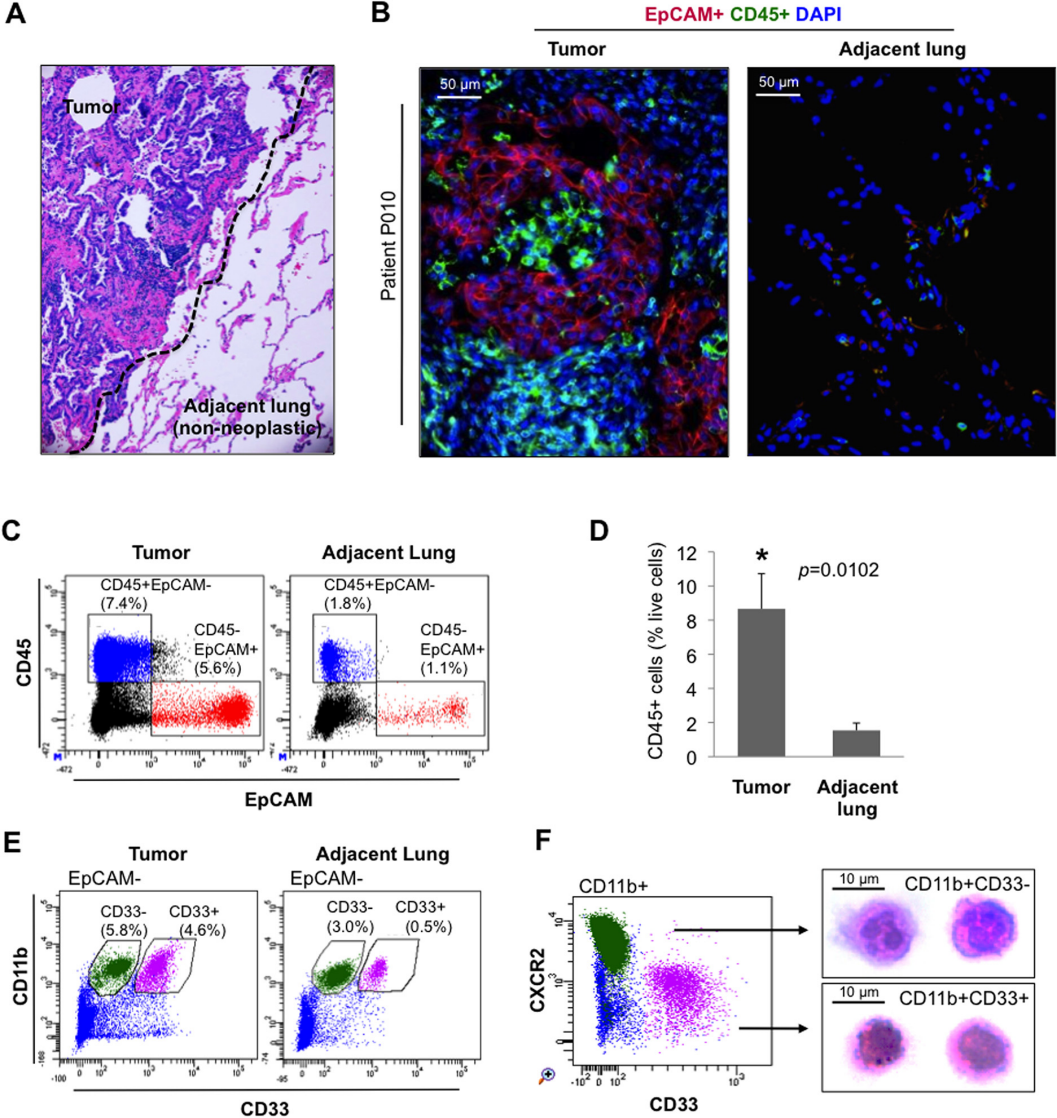
Increased number of bone marrow hematopoietic cells infiltrate lung compared to matched adjacent non-neoplastic lung. (A) H&E staining of lung tissue from an adenocarcinoma patient. The dotted line separates the tumor from the adjacent non-neoplastic lung. Image is at 20X magnification. (B) Representative immunofluorescence image of tumor and matched adjacent non-neoplastic lung of adenocarcinoma patient stained for epithelial cells (EpCAM^+^, red) and BM-derived hematopoietic cells (CD45^+^, green). DAPI (blue) was used to label cell nuclei. (C) Flow cytometry scatter plots showing CD45^+^EpCAM^-^ BM hematopoietic cells and CD45^-^EpCAM^+^ epithelial cells in tumor and matched adjacent lung. (D) Quantitation of CD45^+^EpCAM^-^ BM-derived hematopoietic cells in NSCLC patients (n = 5). Data represents mean ± SEM. (E) Flow cytometry scatter plots showing EpCAM^-^CD11b^+^CD33^-^ BM-derived neutrophils and EpCAM^-^CD11b^+^CD33^+^ BM-derived immature myeloid cells in tumor and matched adjacent lung. (F) Flow cytometry scatter plots showing CD11b^+^CD33^-^ neutrophils are CXCR2^+^ while CD11b^+^CD33^+^ immature myeloid cells are CXCR2^-^ (left panel). Microscopy of flow cytometry sorted cells stained with H&E showing nuclear morphology CD11b^+^CD33^-^ and CD11b^+^CD33^+^ cells (right panel).

We used surgically resected specimens from NSCLC patients, and performed immunohistochemical (IHC) staining to identify regions of adenocarcinoma and adjacent non-neoplastic lung (adjacent lung) (Fig 1A). To determine the relative contribution of BM–derived stromal cells, we stained the adenocarcinoma and adjacent lung with antibodies specific for hematopoietic cells (CD45^+^) and epithelial cells (epithelial cell adhesion molecule, EpCAM^+^) (Fig 1B and S1 Fig). An increased number of CD45^+^ hematopoietic cells were commonly observed in the adenocarcinoma compared to adjacent lung tissue (approximately 5 cm away from the tumor margins; Fig 1B and S1 Fig). Consistently, flow cytometry analysis showed an increase in CD45^+^ hematopoietic cells (>4-folds) in the tumor parenchyma compared to adjacent lung (Fig 1C and 1D).

The CD45^+^ hematopoietic population is heterogeneous and comprised of various subpopulations including T lymphocytes, B cells, and cells of the myeloid lineage [36]. Of the hematopoietic cells, we were interested in cells of the myeloid lineage as these are globally altered in cancer and promote tumor growth by stimulating tumor angiogenesis, suppressing tumor immunity, and promoting metastasis to distant sites [37]. Of the myeloid cells, we focused on immature monocytic myeloid cells (IMMC, CD11b^+^CD33^+^) and neutrophils (CD11b^+^CD33^-^) [38] (Fig 1E). The identities of these myeloid cells were further confirmed by analyzing the expression of other myeloid cell markers and cell nuclear morphology. The CD11b^+^CD33^+^ immature myeloid cells were devoid of CXCR2, where as the CD33^-^ cells were CXCR2^+^ neutrophils (Fig 1F, Left panel). Consistent with flow cytometry data, nuclear morphology analysis showed that the CD11b^+^CD33^+^CXCR2^-^ cells were mononuclear, whereas the CD11b^+^CD33^-^CXCR2^+^ cells were polymorphonuclear (Fig 1F, Right panel). Further evaluation showed that the CD33^+^ IMMCs expressed low levels of macrophage marker CD68 and showed a typical mononuclear scatter pattern (S2A and S2B Fig). The increased prevalence of these specific BM-derived sub-populations in the adenocarcinoma compared to matched adjacent lung tissue suggested that these cells may contribute to NSCLC growth.

### Specific depletion of myeloid cells, such as neutrophils, impairs NSCLC growth in mice

Murine, myeloid subsets are defined as CD11b^+^Gr1^+^ cells, which are comprised of two major subpopulations defined as CD11b^+^Ly6C^high^ (IMMCs) and CD11b^+^Ly6G^high^ (neutrophils) [18, 33, 34]. To assess the effects of depleting specific myeloid cell populations on NSCLC progression, we used a Ly6G-specific monoclonal antibody which has previously been used for depleting neutrophils in mice [35]. We did not deplete Ly6C^high^ cells, as robust reagents for specifically depleting these cells are unavailable.

**Fig 2:**
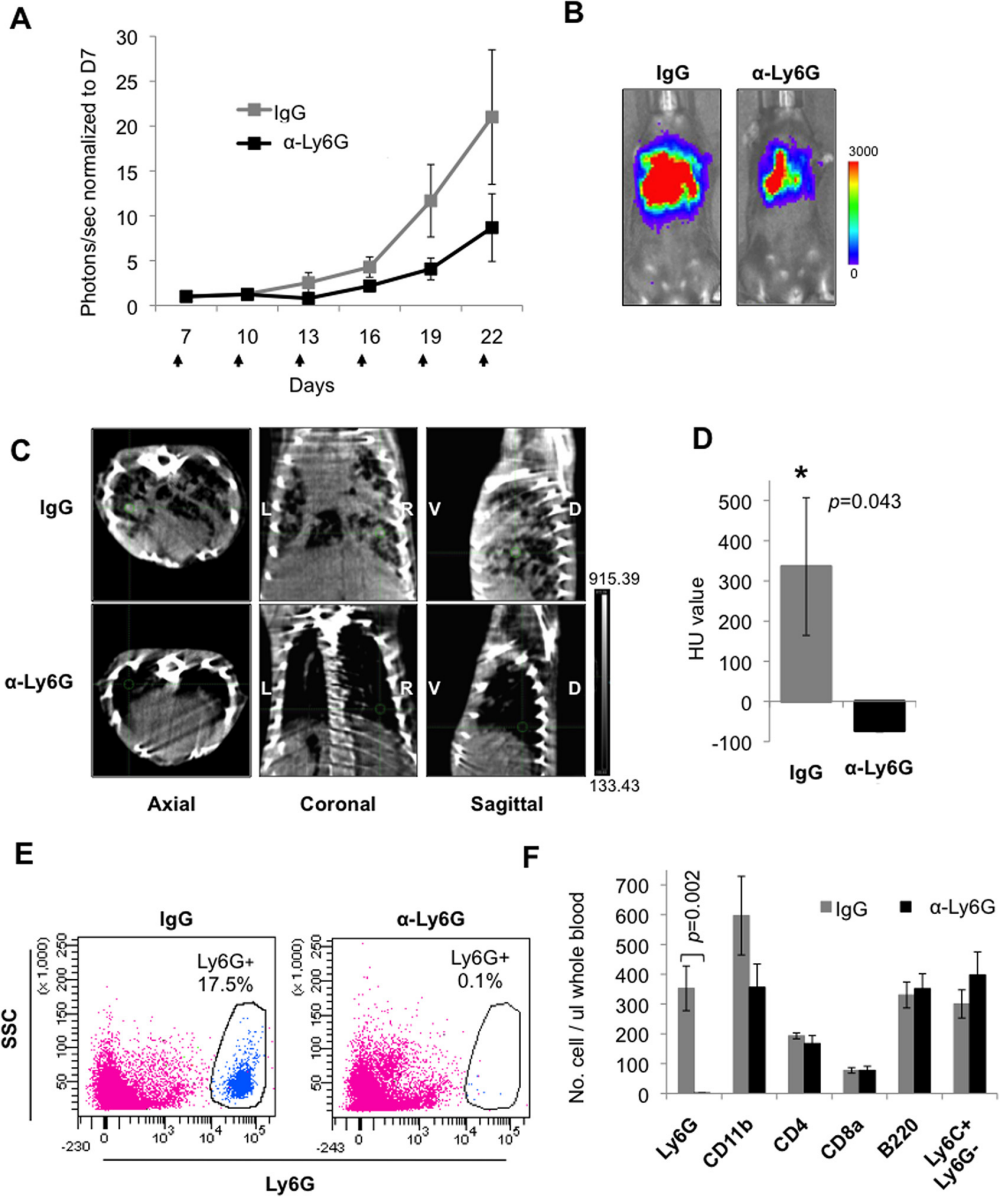
Depletion of Ly6G^+^ neutrophils impair growth of lung adenocarcinoma in mice. (A) Quantitation of orthotopic lung tumors in mice treated with either anti-Ly6G (αLy6G) or control (IgG) antibodies as assessed by bioluminescence (BLI) measurements as a function of time (in days, n = 7 and 6 per group respectively). Arrows indicate days at which antibody was administered. Data represent mean ± SEM. (B) Representative BLI images of animals. Color scale bar depicts the photon flux (photons per second) emitted from these mice at day 22. (C) Representative micro-CT slice images of lungs showing tumors in mice treated with anti-Ly6G or control IgG antibody at day 25. Axial, coronal, and sagittal views shown. Bright objects are high density (bone) and black represents air voids within the animal. Axial images were taken from same relative position in each animal; cross-hair points to the same location in all views. D, dorsal; V, ventral; L, left; R, right. (D) Quantification of lung tumor burden by micro-CT analysis (n = 3 per group) HU, Hounsfield Unit (where -1000 is air, -700 is lung, and +100 to +300 is soft tissue). Data represent mean ± SEM. (E) Flow cytometry scatter plots of peripheral blood showing depletion of Ly6G^+^ cells in anti-Ly6G treated mice as compared with control IgG treated mice at 3 days post-treatment. (F) Flow cytometry analysis of peripheral blood showing numbers (cells per ul of blood) of Ly6G^+^ and CD11b^+^ myeloid cells, CD4^+^ and CD8^+^ T cells, B220^+^ B cells, and Ly6C^+^Ly6G^-^ monocytes. n = 4 per group. Data represent mean ± SEM.

Using the *K-ras* ^G12D^ *p53* ^flox/flox^ mouse model [39] of orthotopic NSCLC, we found that treatment with an anti-Ly6G antibody showed impaired NSCLC growth compared to controls, as determined by whole body bioluminescence imaging (BLI) (Fig 2A and 2B). Micro-computed tomography (micro-CT) revealed a significant decrease in the size of lung nodules in anti-Ly6G treated mice compared to that of IgG control antibody treated mice (Fig 2C), as quantified in Fig 2D. Flow cytometry analysis of peripheral blood confirmed efficient and specific depletion of Ly6G^+^ cells while other major hematopoietic populations remained unperturbed (Fig 2E and 2F, and S3 Fig), suggesting that tumor suppression had occurred due to Ly6G^+^ cell depletion.

### RNA-deep sequencing reveals differentially regulated genes and mRNA isoforms in intratumoral BM myeloid cells

After establishing the critical requirement of myeloid cells for lung cancer progression, we next assessed the activation state of these cells by comparing the transcriptomic profiles of individual specific cell populations taken from within the adenocarcinoma, and also from adjacent lung tissue. For sample preparation and analysis we isolated stromal cells directly from fresh clinical samples without expanding cells in culture, to minimize aberrant culture-induced alterations in cellular and molecular phenotypes. To avoid the introduction of potential bias in transcript distribution, RNA was not amplified. Finally, in each individual patient we restricted our analysis to homogenous stromal populations to unravel cell-specific gene signatures, which could be missed when heterogeneous populations are used in routine bulk tumor profiling. Thus, we did not isolate cell types such as fibroblasts for which homogenous populations cannot be isolated using existing methods.

**Fig 3:**
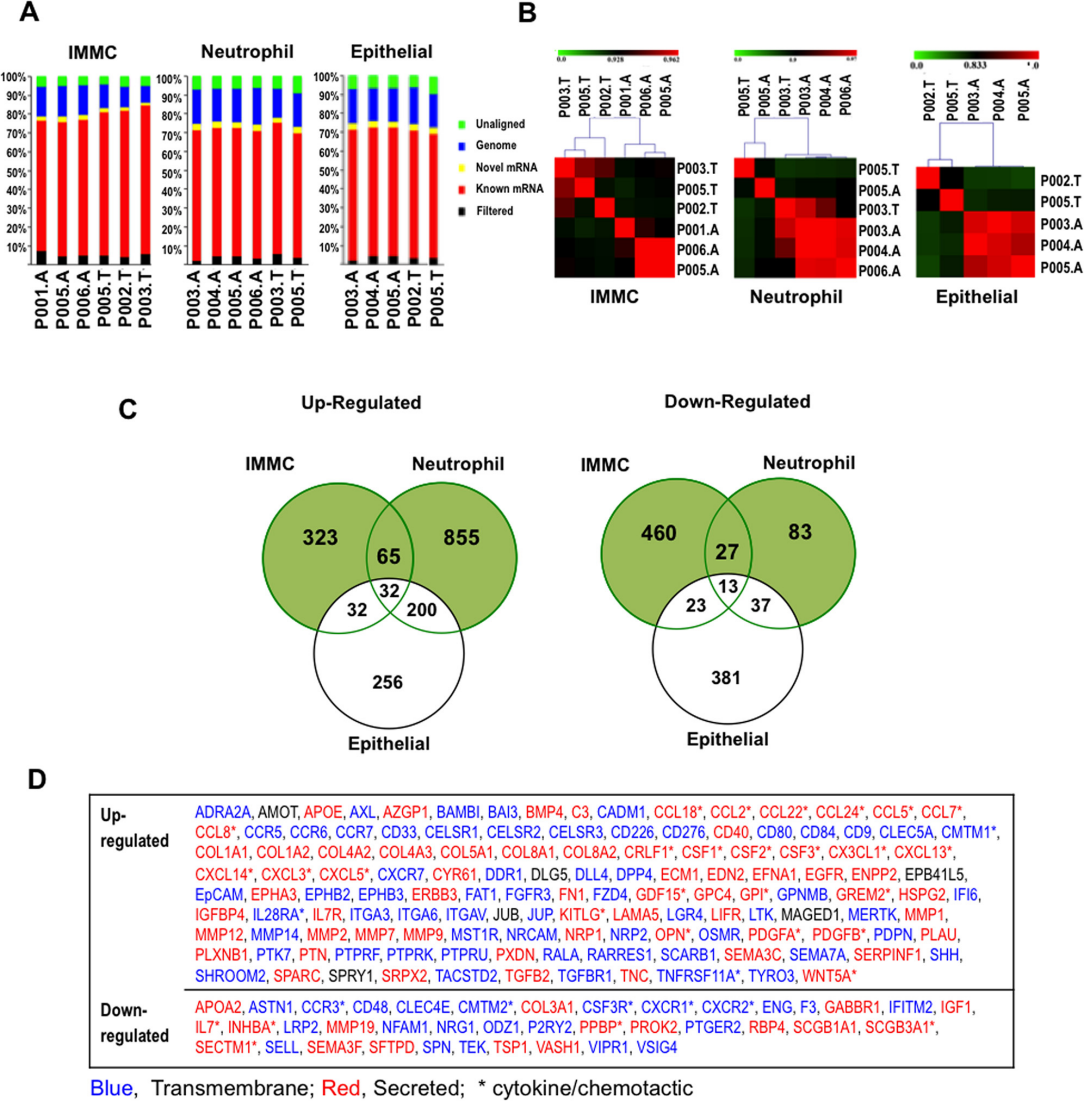
RNA-seq analysis of BM immature monocytic myeloid cells and epithelial cells from NSCLC patients and controls. (A) Summary of RNA-seq reads from adenocarcinoma (tumor)- and adjacent lung-derived IMMCs (immature monocytic myeloid cells), neutrophils, and epithelial cells isolated from 6 adenocarcinoma patients and mapped to known human mRNA, genome, and novel mRNA of Aceview gene model. P, unique patient identifier; A, adjacent non-neoplastic lung tissue; T, neoplastic tumor tissue. (B) Spearman correlation analysis showing clustering of stromal cells derived from IMMCs, neutrophils, and epithelial cells based on global RNA-seq gene expression profiles into distinct tumor and adjacent lung groups. P, unique patient identifier; A, adjacent non-neoplastic lung tissue; T, neoplastic tumor tissue. (C) Venn diagrams showing total number of differentially expressed genes in immature IMMCs, neutrophils, and epithelial cells from adenocarcinoma compared to non-neoplastic adjacent lung. Cutoff of at least 50 unique mapped reads and FDR <5%. The genes in the list show differential expression with p<0.05, and fold change >2. (D) Differentially regulated stromal genes from (C), enriched for potential paracrine functions as determined by Gene Ontology annotation as secreted, extracellular space, or membrane (except membranes of organelles including golgi and endoplasmic reticulum). Of these, genes with the functions in key tumorigenic pathways including angiogenesis, ECM breakdown, cell migration, proliferation, invasion, cytokine function, chemokine function, and chemotactic function were selected. Genes selected for analysis are denoted in blue, transmembrane; red, secreted.

We sorted pure populations of IMMCs, neutrophils, and epithelial cells (ranging from 14.5K-200K cells per population, per patient) from freshly harvested tumors and adjacent lung of stage I-III lung adenocarcinoma patients. RNA from individual cell populations was used to construct cDNA libraries (S4 Fig), which were sequenced using Illumina HiSeq2000 sequencers. To obtain statistically significant gene expression changes and determine patient-to-patient variability, RNA-seq was performed on the same individual sorted cells from the adenocarcinoma and adjacent non-neoplastic lung tissue of six patients (Stage I-III) (S1 Table). RNA-seq data analysis was performed according to our published analysis pipeline [29, 30]. Differential gene expression analysis was performed using the DEseq method [31] which is based on a negative binomial distribution model (S5 Fig). Approximately 80% of the reads aligned to known and novel mRNAs in each case, confirming high quality mRNA sequencing data (Fig 3A). Uniquely mapped reads were used to identify differentially expressed genes (p ≤ 0.05). Importantly, correlation analysis of these genes showed discrete clustering of stromal cells from tumors and adjacent lungs with minimal patient-to-patient variability (Fig 3B), suggesting that RNA-seq data from this patient cohort was sufficiently robust to identify differentially regulated genes. Next, genes differentially regulated in the intratumoral stromal subsets and epithelial cells, compared with the same subsets from adjacent lung tissue, were identified using standard statistical methods with a false discovery ratio (FDR) <5% (Fig 3C). Ingenuity pathway analysis of differentially regulated genes from stromal compartments showed that a major proportion of genes were involved in cancer-specific pathways (data not shown).

Analysis of individual stromal populations identified potential differentially regulated compartment-specific genes, which were filtered using specific criteria to select and prioritize genes for further validation. The criteria used included the selection of genes differentially regulated in specific tumor stromal cell compartments and not in tumor epithelial cells as our goal was to identify stroma-specific targets (Fig 3C), and Gene Ontology (GO) enrichment analysis to identify genes with paracrine activity to capture potential tumor-stroma crosstalk. Using these criteria, approximately 80–100 genes were selected for each cell type (Fig 3D and S2 Table).

**Fig 4:**
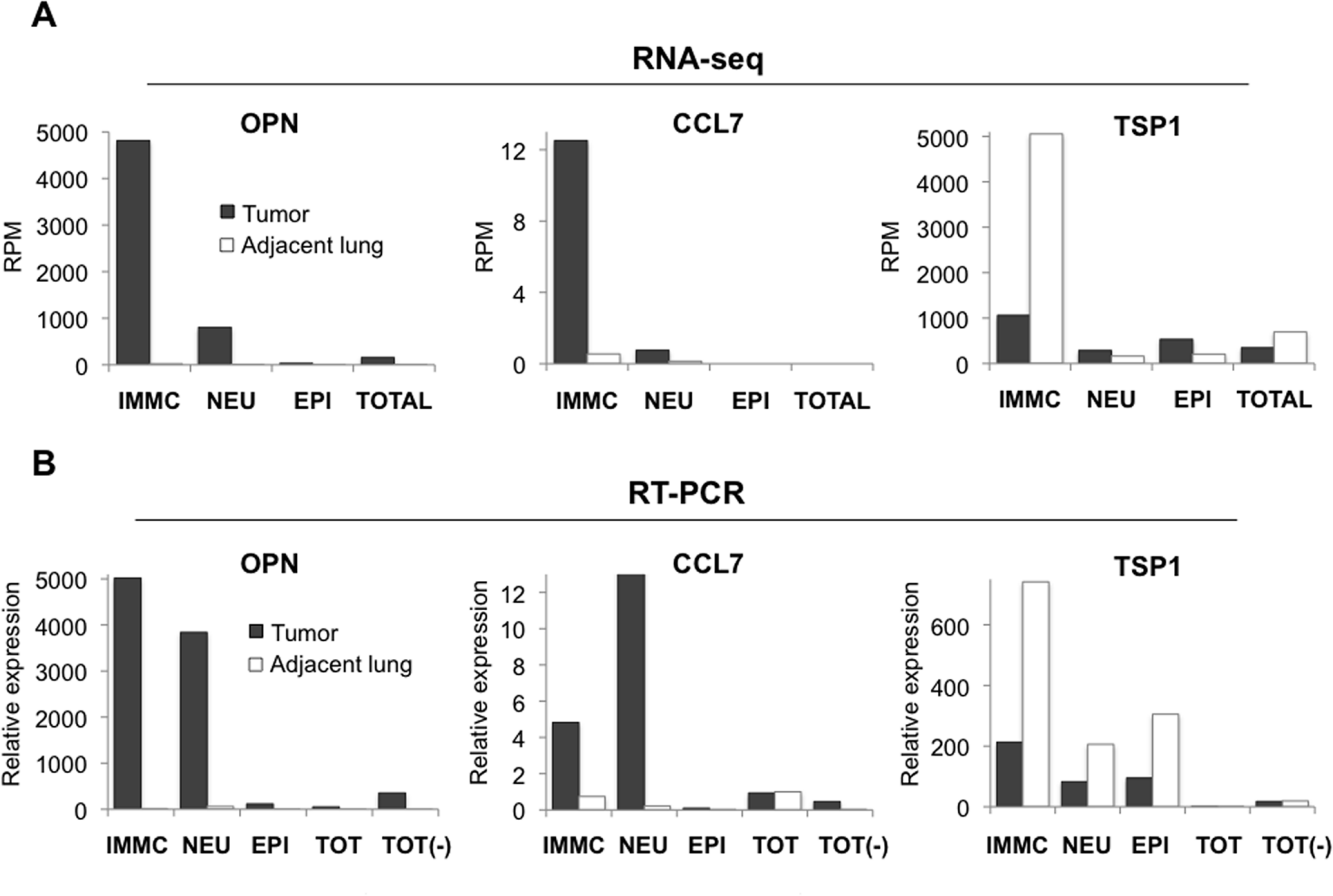
RNA-deep sequencing analysis unravels differentially regulated genes in intratumoral BM-derived myeloid cells. (A) RNA-seq analysis and (B) qRT-PCR validation showing stromal compartment-specific expression of candidate genes *OPN*, *CCL7*, *and TSP1* in cells from the tumor bed compared to adjacent lungs. IMMCs; immature monocytic myeloid cells; NEU, neutrophils; EPI, epithelial cells; TOT, total cells from lungs; Total (-), all stromal cells without IMMCs and neutrophils. Average RPM (reads per million) values are indicated.

Differentially regulated genes in each of the stromal cell types, epithelial cells, or both, were identified (S6 Fig). Notably, bulk tissue analysis (“Total” population, S6 Fig) failed to identify differential regulation of many of these stromal genes further highlighting the advantages of our unique approach of evaluating sorted cell populations from heterogeneous tumor tissue, and suggesting why many of these genes with stromal-specific expression patterns had remained undiscovered in previous whole tumor profiling studies. Candidate genes including *OPN*, *CCL7*, *CLEC5A*, *and GPNNB* were upregulated in IMMCs or neutrophils and not in the epithelial cells (Fig 4A and S6A Fig). On the other hand expression of some genes including *TSP1*, a potent antitumorigenic factor, were downregulated in intratumoral stromal cells (Fig 4A).

**Fig 5:**
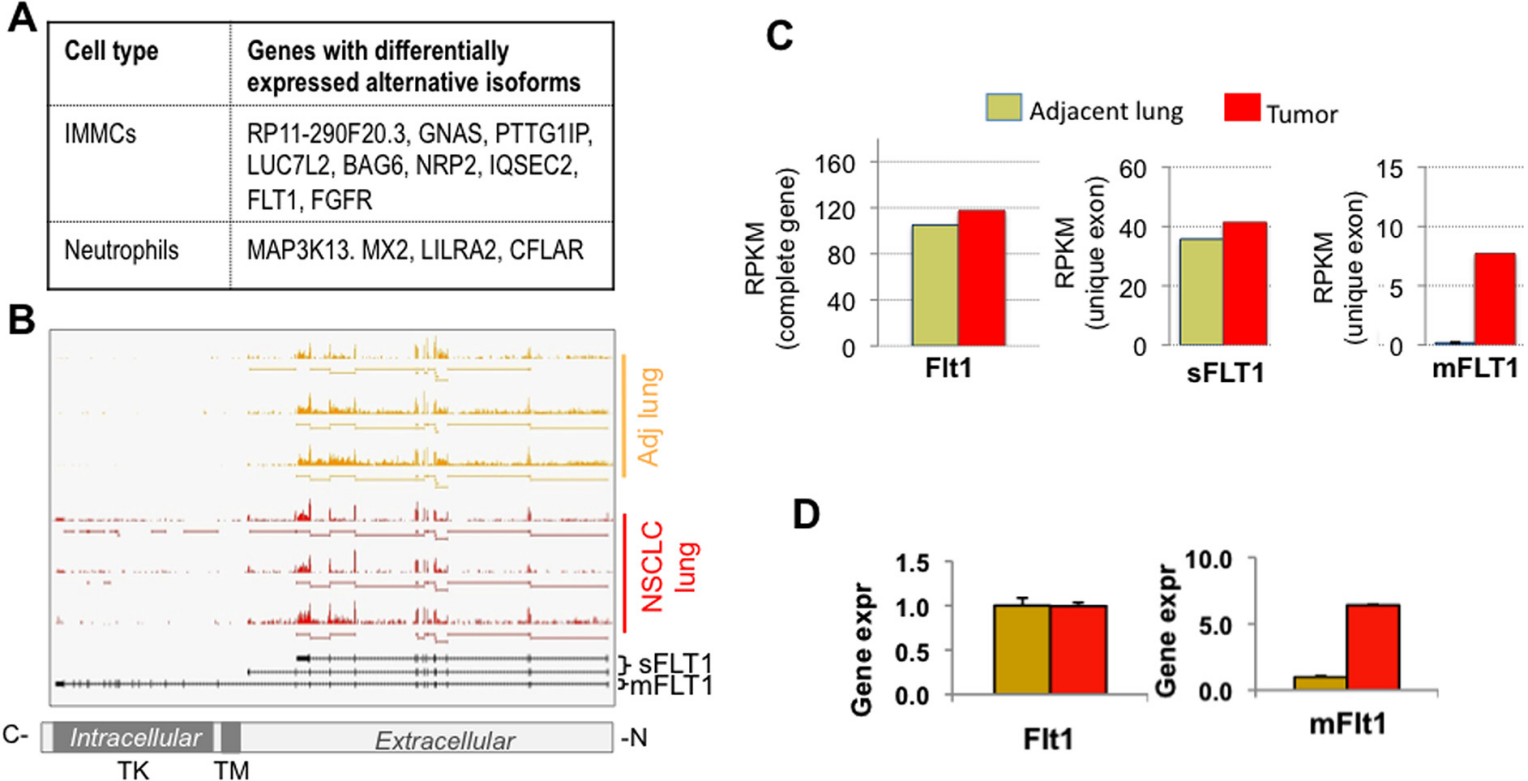
RNA-deep sequencing analysis unravels differentially regulated mRNA isoforms in intratumoral BM-derived myeloid cells. (A) List of stroma-specific genes differentially regulated at the mRNA isoform level. (B) Wiggle plots showing read coverage across the Flt-1 gene in IMMCs from adenocarcinoma of lung (n = 3 patients) and IMMCs form adjacent lungs (n = 3 patients). The status of sFLT1 and mFLT1 is shown. (C) RNA-seq analysis showing *FLT-1* isoform expression levels of total *FLT1*, soluble FLT1 (*sFLT1*), and membrane binding FLT1 (*mFLT1*) in myeloid cells sorted from adjacent lung and tumor. (D) RT-PCR validation of *FLT1* and *mFLT-1* isoform expression in myeloid cells sorted from adjacent lung and tumor.

Next we considered the possibility that for some genes not showing differential expression, their overall transcript abundance could be similar while their various isoforms may be differentially regulated. Indeed, isoform-specific expression of genes such as *VEGF*, *p63*, *p73*, and *CD44* has been correlated with discrete pathologies and survival in lung cancer patients [40–42]. Using an alternative-exon inclusion ratio-based approach to identify the alternative first, alternative last, and skipped exons [29, 30, 43] from the RNA-seq reads, we identified stromal-specific genes regulated at the isoform level (Fig 5A). Among the genes potentially regulated at the isoform level we focused on *FLT-1* (*VEGFR1*) (Fig 5B, 5C and 5D) as the *VEGF*-*FLT-1* axis constitutes a major regulator of angiogenesis. It has been shown that *FLT-1* mRNA is alternatively spliced to encode both a full-length receptor tyrosine kinase (*mFLT-1*) that is proangiogneic, and a soluble isoform (*sFLT-1*) that binds and sequesters *VEGF* and *PLGF* [44] and is therefore antiangiogenic. As determined by the RNA-seq analysis, the overall expression of *FLT-1* or *sFLT-1* was similar in the CD33^+^ IMMCs from tumors and adjacent lung tissue, however the *mFLT*-1 was specifically upregulated in tumor IMMCs (Fig 5C). Importantly, RT-PCR analysis using isoform-specific primers confirmed *mFLT-1* expression levels in the intratumoral IMMCs (Fig 5D). Notably, *FLT-1* expression in cells of the myeloid lineage has been shown to regulate *VEGF*/*PLGF*-oriented migration, survival, and production of angiogenic factors [45, 46], which can promote tumor growth [47], suggesting that it may have a protumorigenic function in NSCLC.

### Validation of differentially regulated compartment-specific stromal genes

As our goal was to identify differentially regulated genes in intratumoral stromal cells, we validated a subset of the genes selected from the RNA-seq analysis by quantitative RT-PCR (qRT-PCR) to confirm that their differential expression was confined to specific individual intratumoral cellular compartments (IMMCs, neutrophils, and epithelial cells). Included in the qRT-PCR analysis were sorted samples consisting of all stromal cells without IMMCs and neutrophils (“Total (-)” population, Fig 4B), to further confirm the lineage specific expression of these candidate genes.

qRT-PCR analysis showed significant correlation with RNA-seq data not only in the relative expression levels but also in stromal compartment specificity. For example, candidate genes including *OPN*, *CCL7*, *and TSP1* were differentially expressed in IMMCs or neutrophils and not in the epithelial cells or other stromal cells (Fig 4B).

We reasoned that differentially regulated genes identified in intratumoral stromal cells may contribute to carcinogenesis, and went on to select stromal genes including *OPN*, *CCL7*, and *TSP1* as potential candidates as they are secreted and are likely to influence NSCLC progression by conferring paracrine functions on tumor epithelial cells. OPN is a secreted phosphoglycoprotein that has been shown to contribute to tumor progression and metastasis [48]. Increased OPN expression has been observed in human breast, lung, prostate, colon, ovarian, and gastric cancers [49]. CCL7 is a relatively understudied cytokine, however its expression in tumor stroma has been shown to enhance invasion and migration of oral squamous carcinoma cells [50]. CCL7 has also been shown to promote neutrophil recruitment in lung inflammation [51]. TSP1 is a secreted extracellular matrix protein and one of the most potent inhibitors of tumor angiogenesis and growth [52, 53]. Consistent with this tumor suppressive property, *TSP1* expression was found to be downregulated in intratumoral myeloid lineage cells.

**Fig 6:**
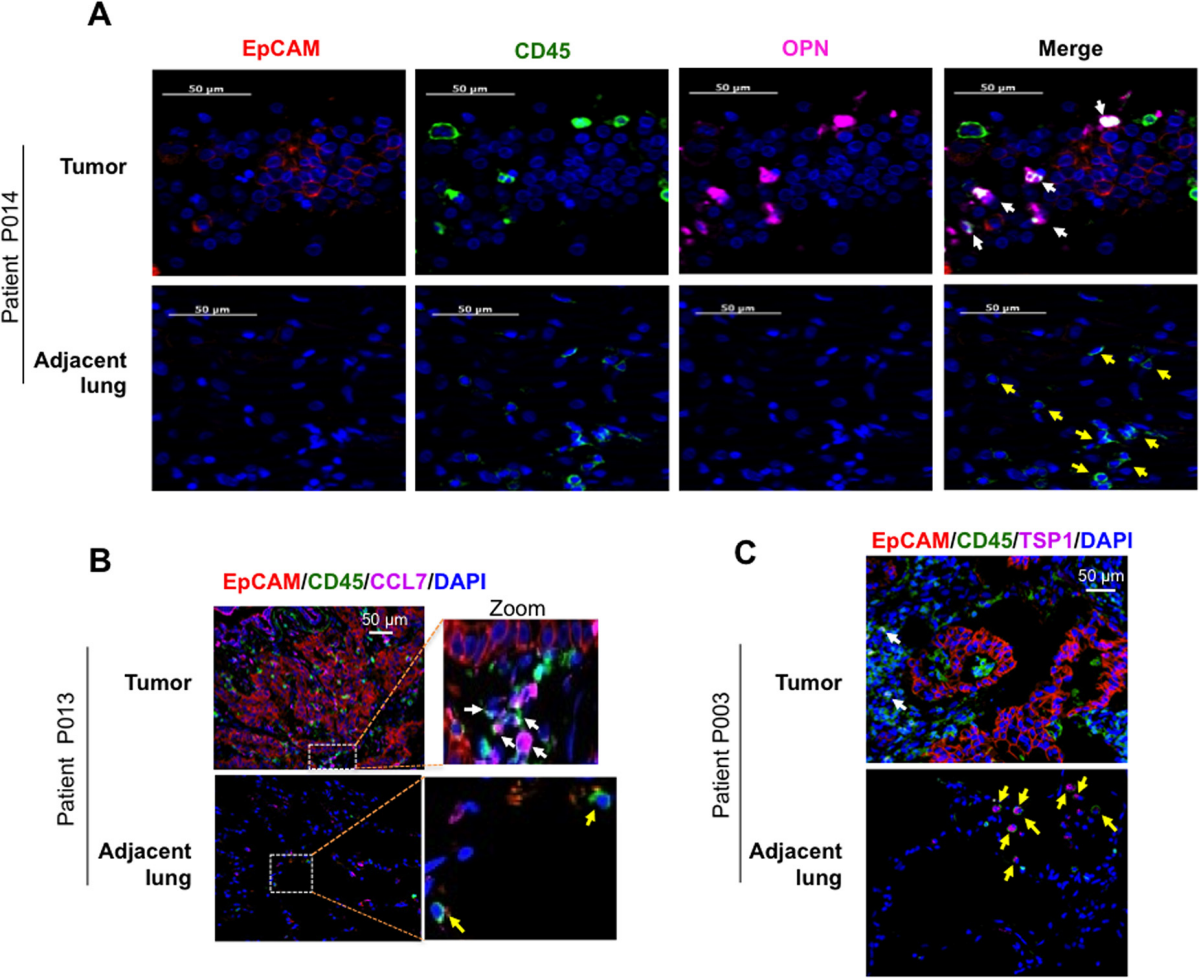
Differential regulation of stromal factors OPN, CCL7, and TSP1 in the intratumoral BM cells. (A) Representative immunofluorescent images showing OPN expression in CD45^+^ BM cells (Upper panel, white arrows) but not in EpCAM^+^ tumor epithelial cells (red). OPN-expressing BM cells were not detected in the matched adjacent non-neoplastic lung tissue (Lower panel, yellow arrows). (B) Representative immunofluorescent images showing CCL7 expression in CD45^+^ BM cells in tumor tissue (Upper panel, white arrows). CCL7 expressing BM cells were not detected in matched adjacent non-neoplastic lung tissue (Lower panel, yellow arrows). (C) Representative immunofluorescent images showing lack of TSP1 expression in CD45^+^ BM cells in tumor tissue (Upper panel, white arrows), and presence of Tsp-1 in BM cells in matched adjacent non-neoplastic lung tissue (Lower panel, yellow arrows).

Using immunofluorescence staining of human lung adenocarcinomas, we further assessed the candidate genes both for expression levels and BM stromal cell specificity. As expected from the RNA-seq and qRT-PCR analysis (Fig 4), OPN staining was confined to a subset of intratumoral CD45^+^ BM stromal cells and not the epithelial cells (Fig 6A, Upper panel). Consistently, stromal cells in the adjacent non-neoplastic lungs did not exhibit increased expression of this gene (Fig 6A, Lower panel). Similar staining patterns were observed in tumor tissue from other adenocarcinoma patients (S7A Fig). Further analysis showed that, of the CD45^+^ hematopoietic cells, the CD11b^+^ myeloid subset were the main contributors of OPN (S7B Fig). Using a similar approach, we found that CCL7 expression was also confined to the intratumoral BM cells compared to adjacent lungs (Fig 6B and S7C Fig). In contrast, expression of TSP1 was suppressed in the intratumoral BM cells compared to adjacent lungs, consistent with the RNA-seq and RT-PCR analysis (Fig 6C and S7D Fig).

### Functional analysis of differentially regulated stromal genes in conferring tumorigenic potential in lung cancer cells

**Fig 7:**
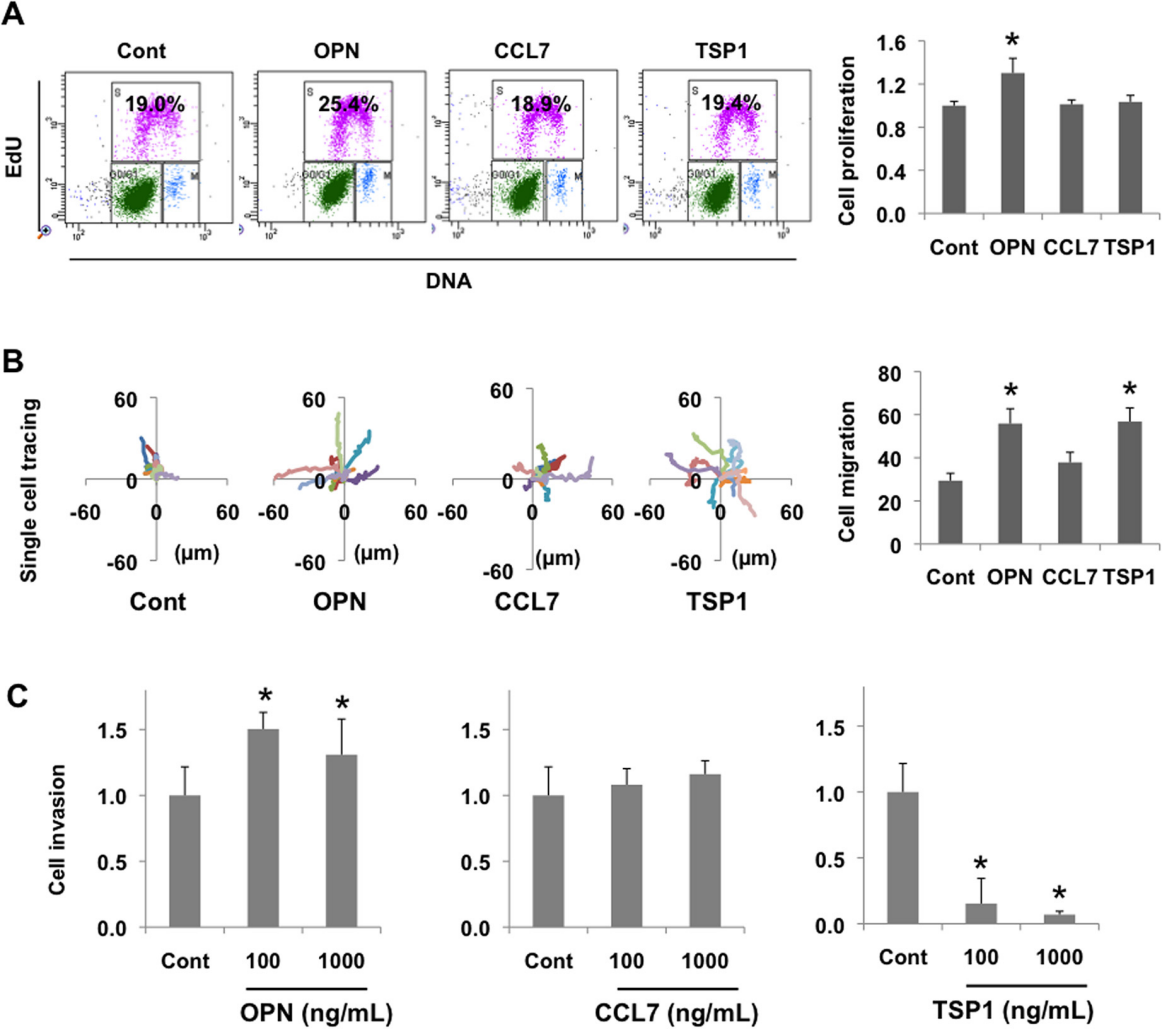
Stromal factors OPN, CCL7, and TSP1 affect key tumorigenic properties of human NSCLC cells. (A) Cell proliferation analysis of H1650 lung cancer cells treated with recombinant human proteins OPN, CCL7, and TSP1 at 100ng/mL. Left panel; the percentage of S phase cells in total cell population was analyzed by flow cytometry, as indicated by numbers. Representative plots are from two independent experiments. Right panel; bar graphs showing quantitation of cell proliferation ratio of factor treated cells as compared with control untreated cells. *p < 0.01. (B) Left panel; Graphs representing tracked movement of single H1650 lung cancer cells (n = 10) by live microscopy imaging following treatment with OPN, CCL7, and TSP1 recombinant proteins (1μg/mL). Each trace represents the migration of a single cell. Right panel; bar graphs showing quantitation of distances cells have moved. *p < 0.01. (C) Cell invasion analysis of H1650 cells in the presence of OPN, CCL7 and Tsp1 at indicated concentrations. Numbers indicate the relative invasion rate as compared with control untreated cells. Representative plots are from two independent experiments. *p < 0.01.

We next posited that differentially regulated stromal-specific genes identified in this study by producing secreted factors might contribute to tumor progression in a paracrine fashion by producing secreted factors. To investigate this, we determined whether these gene products could directly affect key tumorigeneic properties such as proliferation, migration, or invasion of human lung NSCLC cells. Human lung cancer cells (H1650) were treated with varying concentrations of gene-specific recombinant proteins. Flow cytometry analysis showed that OPN treatment increased the percentage of cells in S phase, while CCL7 and TSP1 did not, suggesting that OPN stimulates tumor cell proliferation (Fig 7A). Next, we investigated the paracrine effect of these proteins on cell migration. In cell migration assays using live cell tracking, treatment with OPN, CCL7, and TSP1 (1μg/mL 24 hours) enhanced the mobility of H1650 cells, albeit with different degrees of migration observed (Fig 7B). In a similar way, we determined the potential of these proteins in influencing cell invasion, and observed that while OPN increased invasion, notably, TSP1 significantly inhibited tumor cell invasion through Matrigel-coated membranes (Fig 7C). These results suggest that the stromal-derived factors OPN, CCL7, and TSP1 identified from our RNA-seq analysis affect the malignant properties of human lung cancer cells by promoting either proliferation, migration, or invasion or a combination of these. Clearly, OPN was able to promote invasion, migration, and invasion of lung cancer cells, while CCL7 showed a marginal increase in cell migration and did not affect proliferation or invasion, suggesting that it may possess other protumorigenic properties such as being a chemoattractant in the tumor microenvironment. TSP1 showed robust suppression of cell invasion, as would be expected for an inhibitor of tumorigenesis, however paradoxically it also increased cell migration an observation which has been made in other studies [54, 55].

Taken together, these results suggest that intratumoral stromal cells may contribute to NSCLC growth by expressing elevated levels of the protumorigenic genes OPN and CCL7, and by suppressing the anti-tumorigenic gene TSP1.

## Discussion

Little is known about the contribution and pathophysiological role of stromal cells in NSCLC. Using immunostaining and flow cytometry analysis we have shown increased recruitment of BM-derived stromal cells in tumor tissue compared with adjacent non-neoplastic lung tissue in NSCLC patients. Importantly, we show that subsets of BM-derived stromal cells have a crucial role in NSCLC progression, since specific depletion of neutrophils significantly impaired tumor progression in mice. Indeed, studies show that elevated neutrophil counts correlate with poor clinical outcomes in NSCLC patients [56], suggesting that in NSCLC patients altered activation of recruited BM cells by the tumor epithelial cells may contribute to tumor progression. In this study, we have demonstrated that the analysis of specific individual intratumoral stromal compartments can lead to the identification of differentially regulated genes that may impact the design of novel targeted therapies in NSCLC.

We provide proof-of-concept that it is not only important to separate the stromal component of the tumor when identifying signaling pathways, but that it is critical to isolate specific cell populations from the tumor to fully understand the complex tumor-stromal crosstalk which has the potential to lead to novel prognostic biomarkers and therapeutic interventions. Analysis of the gene expression profiles of myeloid populations in tumor and adjacent tissues revealed approximately 80–100 differentially regulated genes. Among these candidates, expression of OPN was >1000-fold upregulated in intratumoral myeloid cells.

OPN is a secreted phosphoglycoprotein that has been shown to contribute to tumor progression and metastasis [48]. Increased OPN expression has been observed in human breast, lung, prostate, colon, ovarian, and gastric cancers [49]. However, the biological significance of OPN expression in intratumoral myeloid cells has remained unclear. Our finding that in early stage NSCLC OPN is upregulated in intratumoral BM cells signifies the importance of evaluating its potential as a prognostic marker for identifying subsets of NSCLC patients with a high risk of recurrence.

The differentially regulated stromal genes identified in our study have the potential to contribute to NSCLC progression by conferring paracrine functions on tumor epithelial cells. In agreement with this notion, recombinant OPN promoted proliferation, migration, and invasion of lung cancer cells in a paracrine fashion, suggesting that OPN has tumor promoting activity in the microenvironment. Given that multiple and complex mechanisms are involved in the role of OPN in cancer, much remains to be unraveled about these mechanisms and the functional contributions of OPN produced by different cell types in order to pursue anti-OPN therapeutic strategies. For example, specific loss/gain-of-function of OPN in BM-derived myeloid cells in mice would provide insights into its role in stroma-mediated tumor progression. To accelerate clinical translation, further studies are necessary to determine a pharmacological targeting strategy for OPN. Recently developed anti-OPN1 neutralizing monoclonal antibodies, AOM1 [49] and hu1A12 [57], have effectively blocked OPN and impaired breast and lung tumors in mice. In light of our study it will be important to determine whether this anti-tumorigenic phenotype resulted mainly from stroma-specific suppression of OPN. Similarly, in future studies other stromal-specific genes identified in this study should be assessed for prognostic and therapeutic potential. For example, a potent and highly selective humanized antibody inhibitor of MMP14 activity, DX-2400, blocked tumor growth in mouse models of breast and liver carcinogenesis, without causing overt toxicity as has been observed with other MMP inhibitors [58, 59]. Similarly, a CCL7 neutralizing antibody has been shown to inhibit CCL7-induced invasion and migration of oral carcinoma cells [50], and anti-CCL2 and-CCR3 antibodies are also being evaluated [50, 60].

TSP1 is a potent anti-tumorigenic factor. Through interaction with its receptor CD36, which is expressed on endothelial cells, TSP1 inhibits angiogenesis by reducing endothelial cell migration and survival [61]. Therefore, tumors overexpressing TSP1 usually exhibit less angiogenesis, grow slower, and show defects in metastasis. TSP1 is expressed in various cell types including platelets, endothelial cells, stromal fibroblasts, smooth muscle cells, monocytes, and macrophages [62–66]. However, in our study we found that TSP1 expression was confined to myeloid cells in NSCLC. Importantly, TSP1 was downregulated in intratumoral myeloid cells suggesting that restoration of TSP1 levels in the intratumoral stromal cells constitutes a viable strategy to block NSCLC growth. Importantly, TSP1 is also being exploited in clinical trials; and a TSP1 mimetic peptide, ABT-510, which retains the tumor suppressive activity of TSP1 is being used for the treatment of solid tumors, and was found to inhibit tumor growth in the mouse Lewis lung carcinoma model [67].

GSEA revealed downregulated pathways pertaining to immune response and cellular defense response in intratumoral neutrophils, suggesting that the tumor cells affect the proliferation and differentiation of myeloid cells, orchestrating an immune suppressive microenvironment. Further validation and functional analysis of GSEA-identified candidate genes in the NSCLC microenvironment will constitute an area of future investigation.

This study suggests that molecular alterations within the reprogrammed tumor stroma offer novel avenues for therapeutic interventions. Compared to cancer cells, which frequently acquire drug resistance, stromal cells are genetically more stable and therefore less likely to evolve acquired resistance to targeted therapy. It is likely that targeted stromal therapeutics for NSCLC patients may complement conventional treatments that exclusively target cancer cells. In addition to the identification of stromal-specific genes with prognostic and therapeutic potential, our approach of analyzing intratumoral stromal cells will help to define the basic biological mechanisms of the disease, and will open a new field of research for mechanistic and translational studies of lung cancer.

## Supporting Information

S1 Fig Increased number of bone marrow hematopoietic cells infiltrate the adenocarcinoma of NSCLC patients. (TIF)

S2 Fig Validation of sorted IMMCs and neutrophils. (TIF)

S3 Fig Flow cytometry analysis of peripheral blood in mice treated with anti-IgG and anti-Ly6G antibodies. (TIF)

S4 Fig Quality control analysis of RNA harvested from flow cytometry sorted cells from fresh clinical tissue and RNA-seq libraries. (TIF)

S5 Fig RNA-seq data analysis pipeline. (TIF)

S6 Fig RNA-deep sequencing analysis unravels differentially regulated genes in specific intratumoral stromal compartments. (TIF)

S7 Fig OPN, CCL7, and TSP1 are differentially regulated in the intratumoral BM cells. (TIF)

S1 Table Description of lung cancer patients used in this study. (TIF)

S2 Table Differentially regulated genes for each cell type. (TIF)
