# Supplementary figures and images for: Identification of Reprogrammed Myeloid Cell Transcriptomes in NSCLC
Source: PLoS One. 2015 Jun 5;10(6):e0129123. doi: 10.1371/journal.pone.0129123 (PMC4457876; doi:10.1371/journal.pone.0129123)

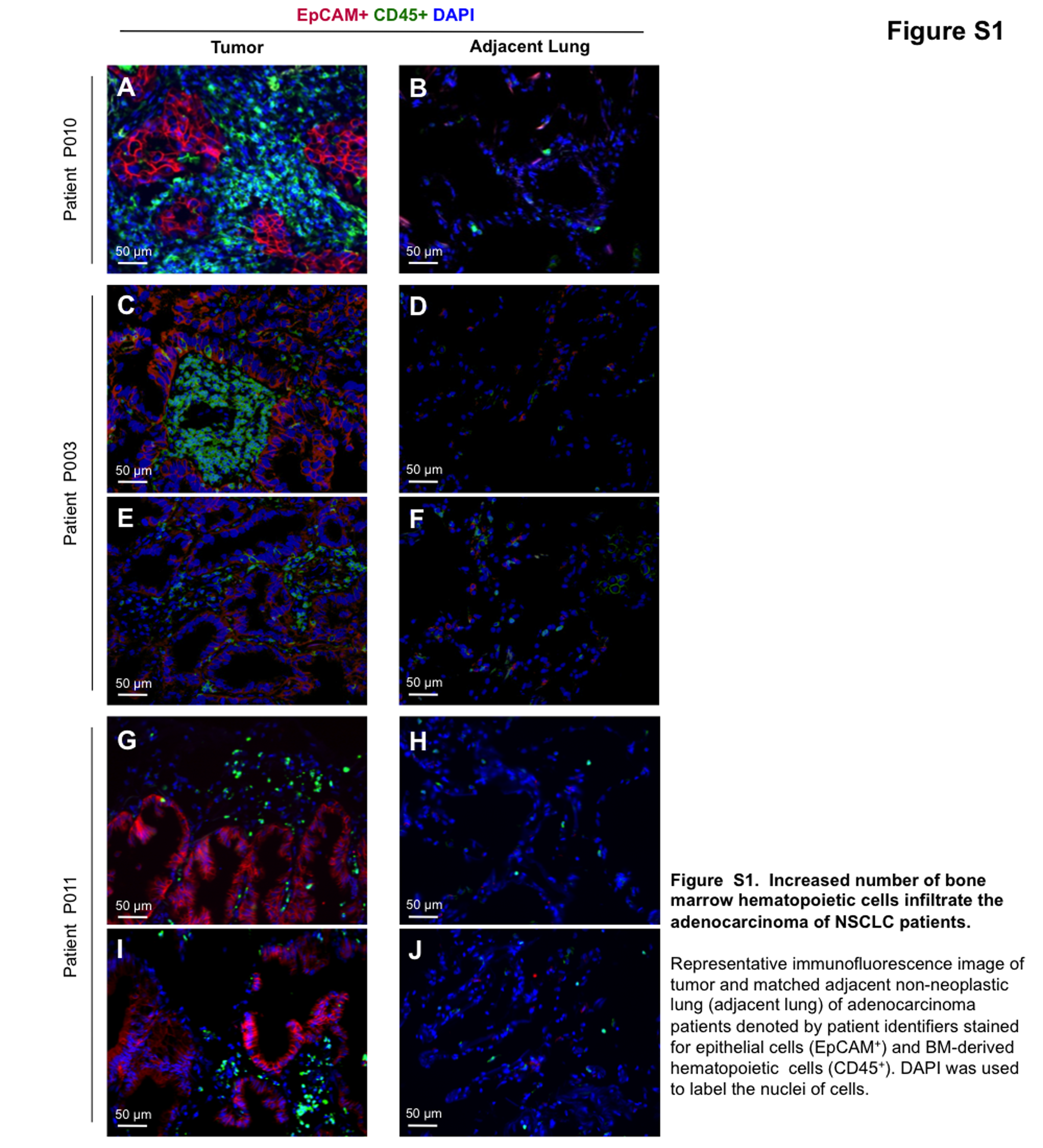

Supplement: S1 Fig — (TIF) [file pone.0129123.s001.tif]

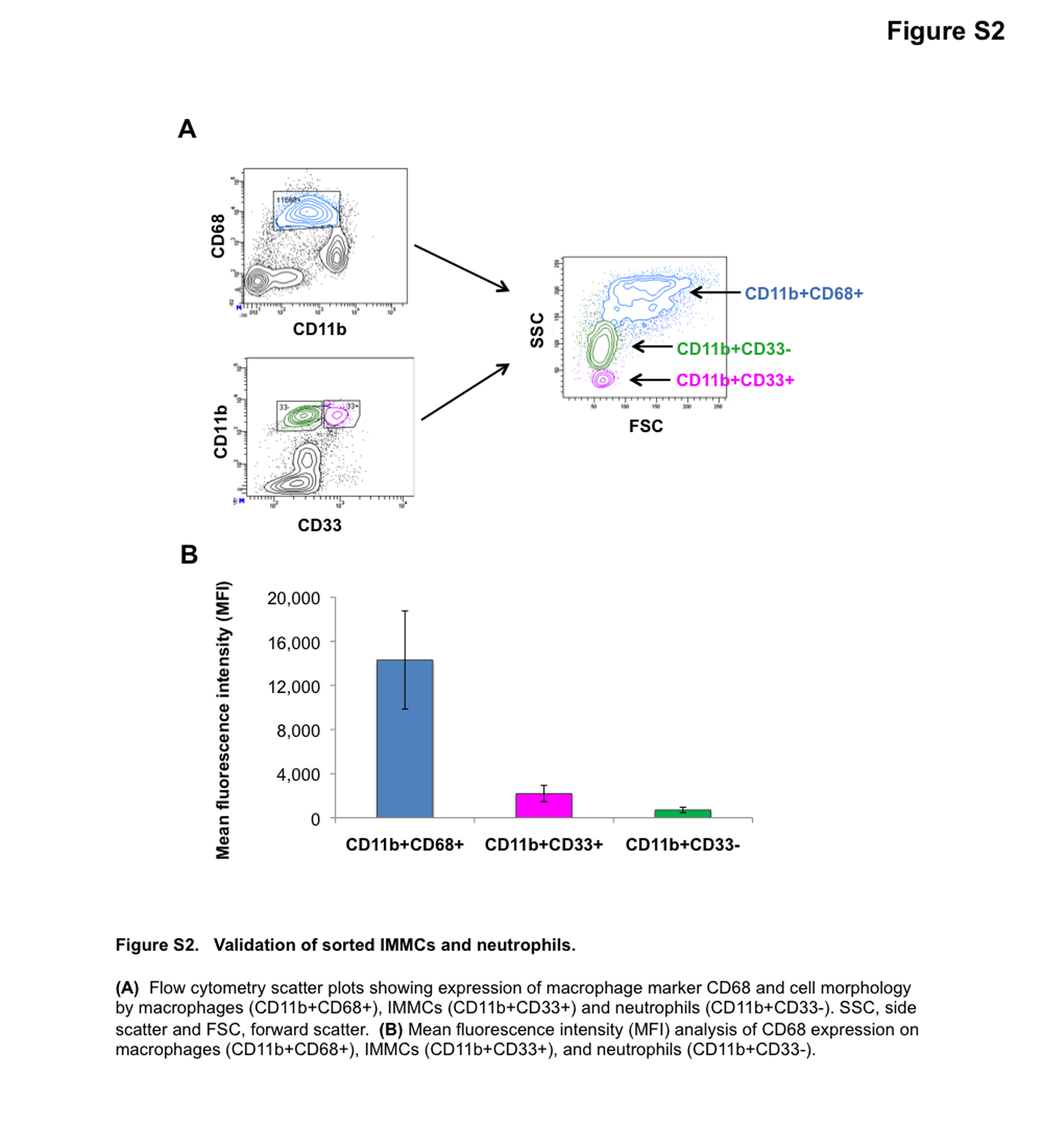

Supplement: S2 Fig — (TIF) [file pone.0129123.s002.tif]

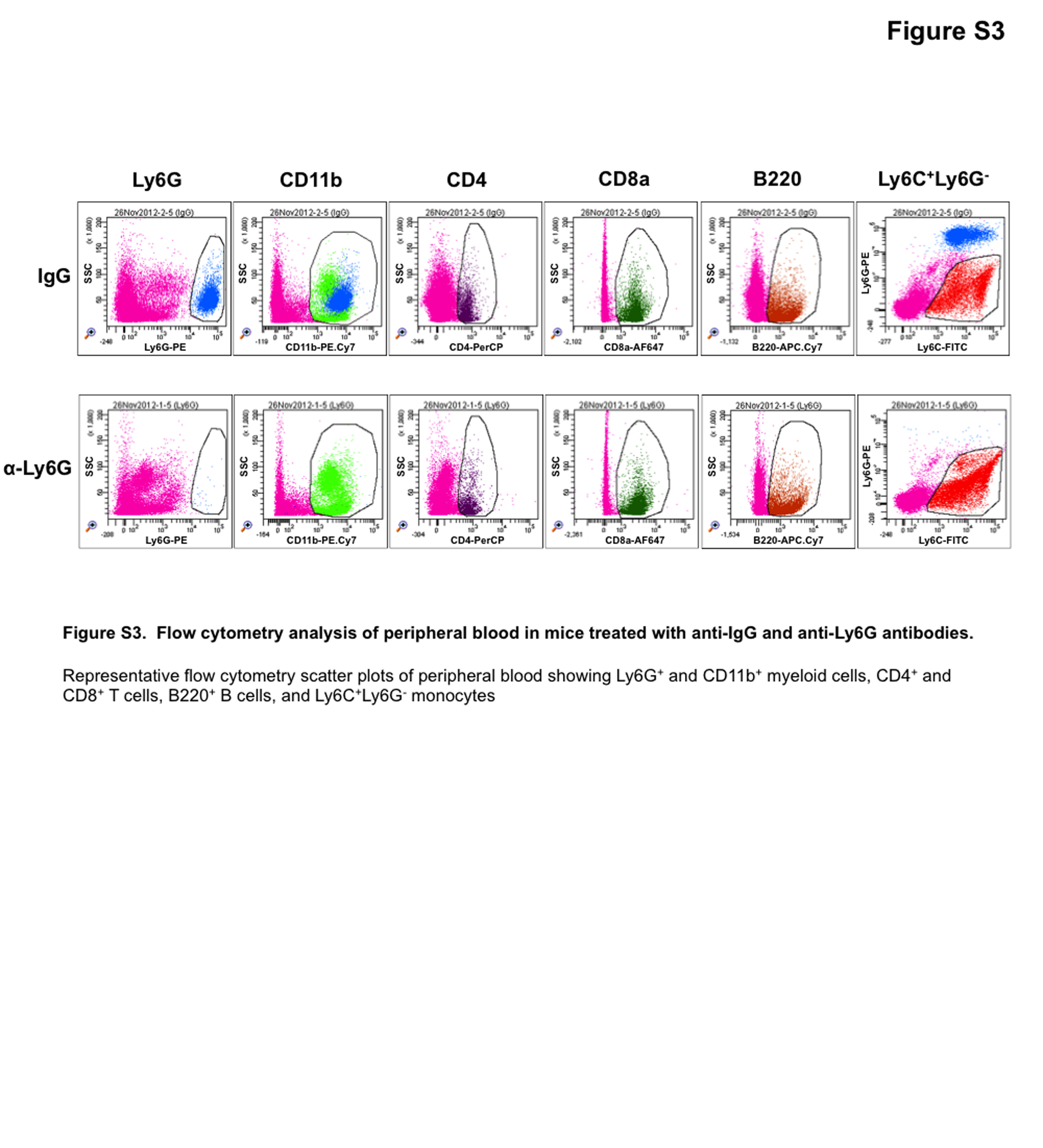

Supplement: S3 Fig — (TIF) [file pone.0129123.s003.tif]

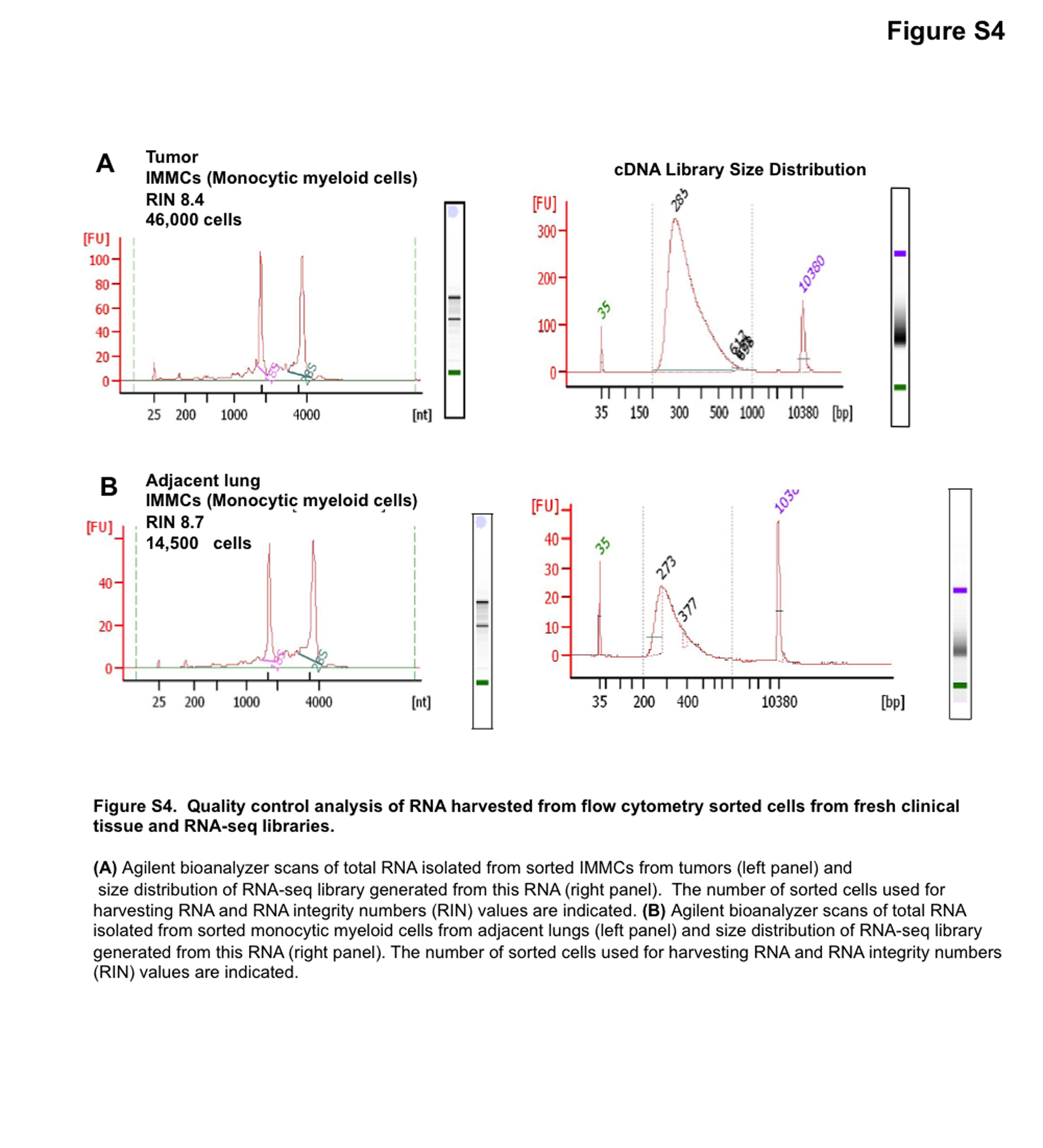

Supplement: S4 Fig — (TIF) [file pone.0129123.s004.tif]

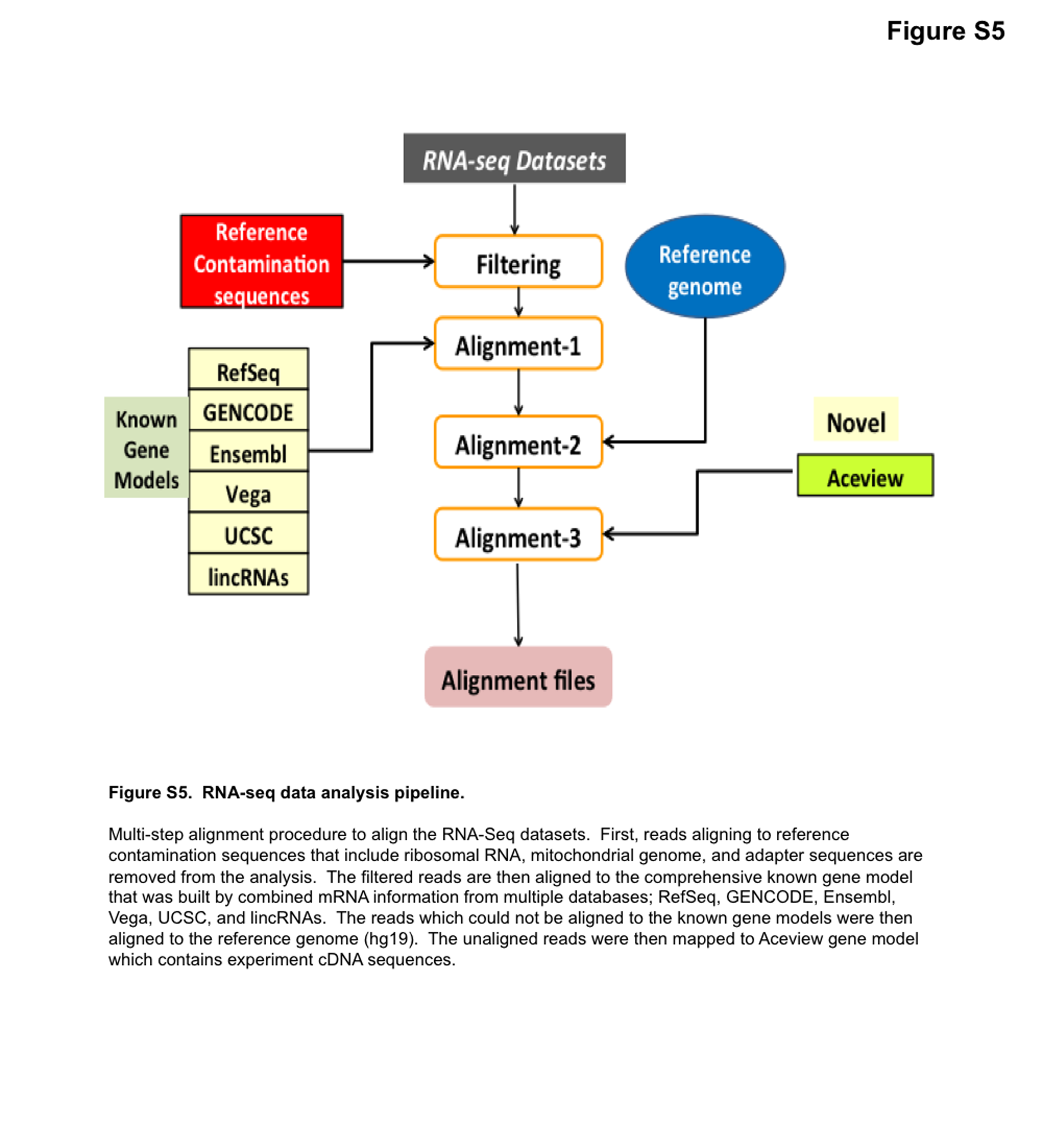

Supplement: S5 Fig — (TIF) [file pone.0129123.s005.tif]

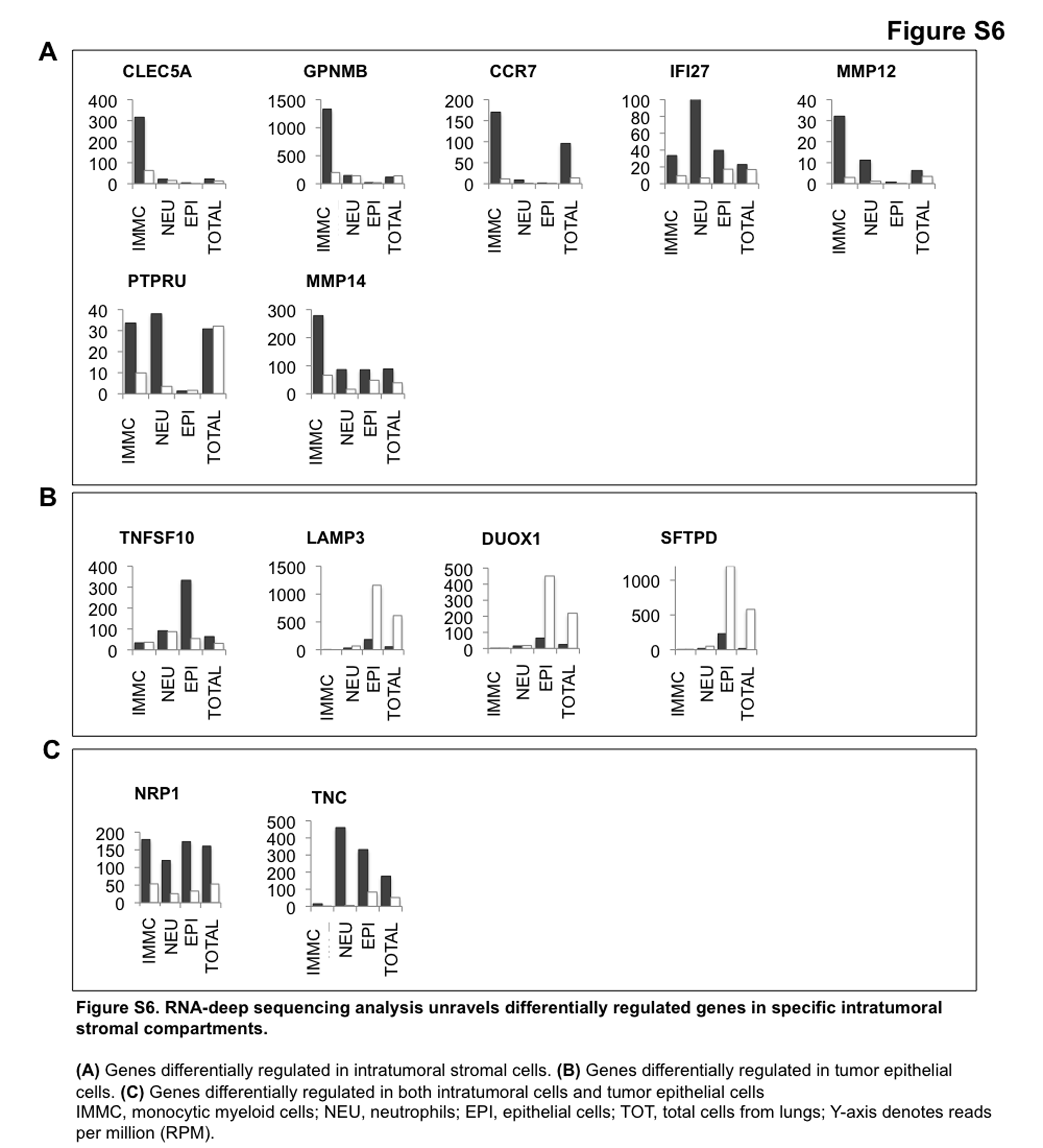

Supplement: S6 Fig — (TIF) [file pone.0129123.s006.tif]

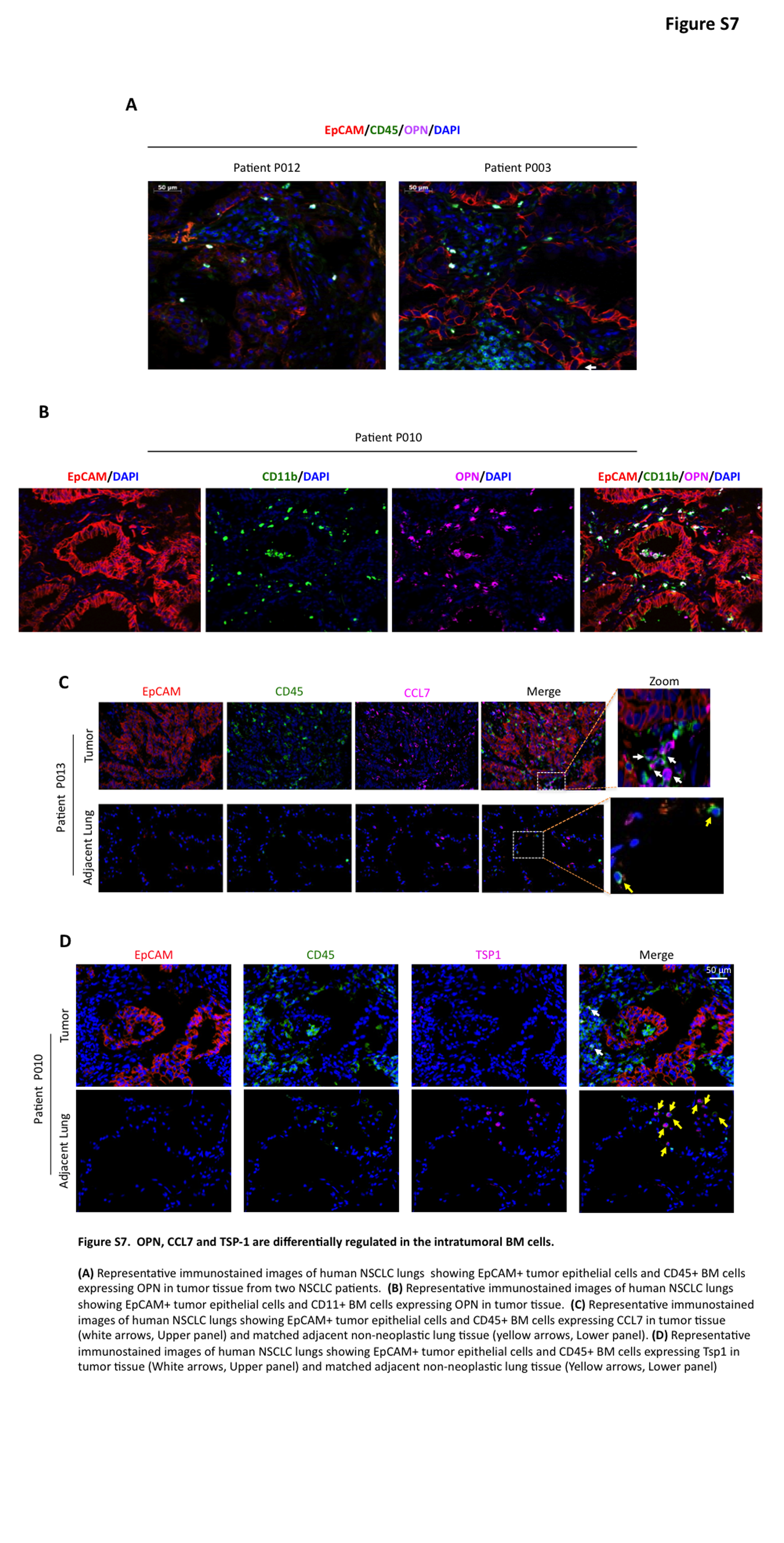

Supplement: S7 Fig — (TIF) [file pone.0129123.s007.tif]

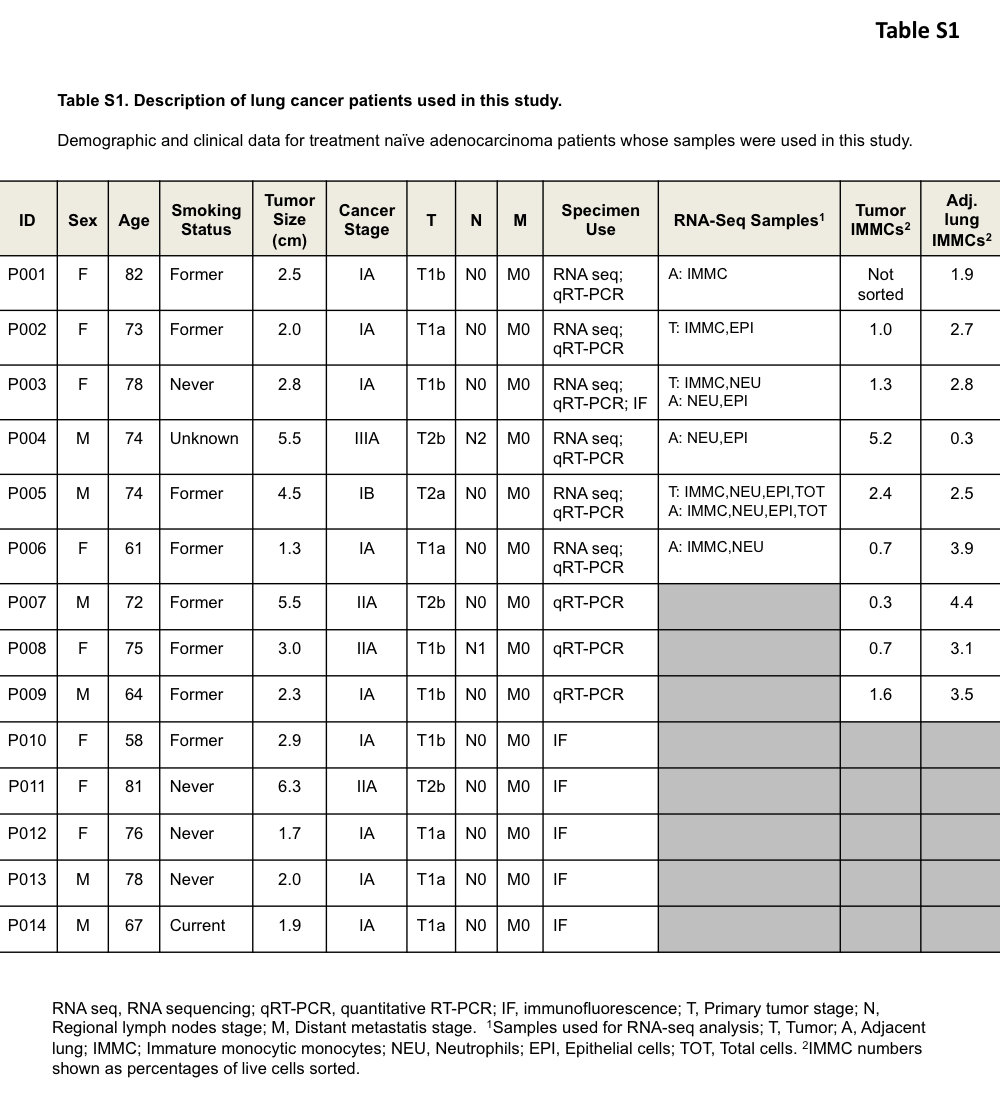

Supplement: S1 Table — (TIF) [file pone.0129123.s008.tif]

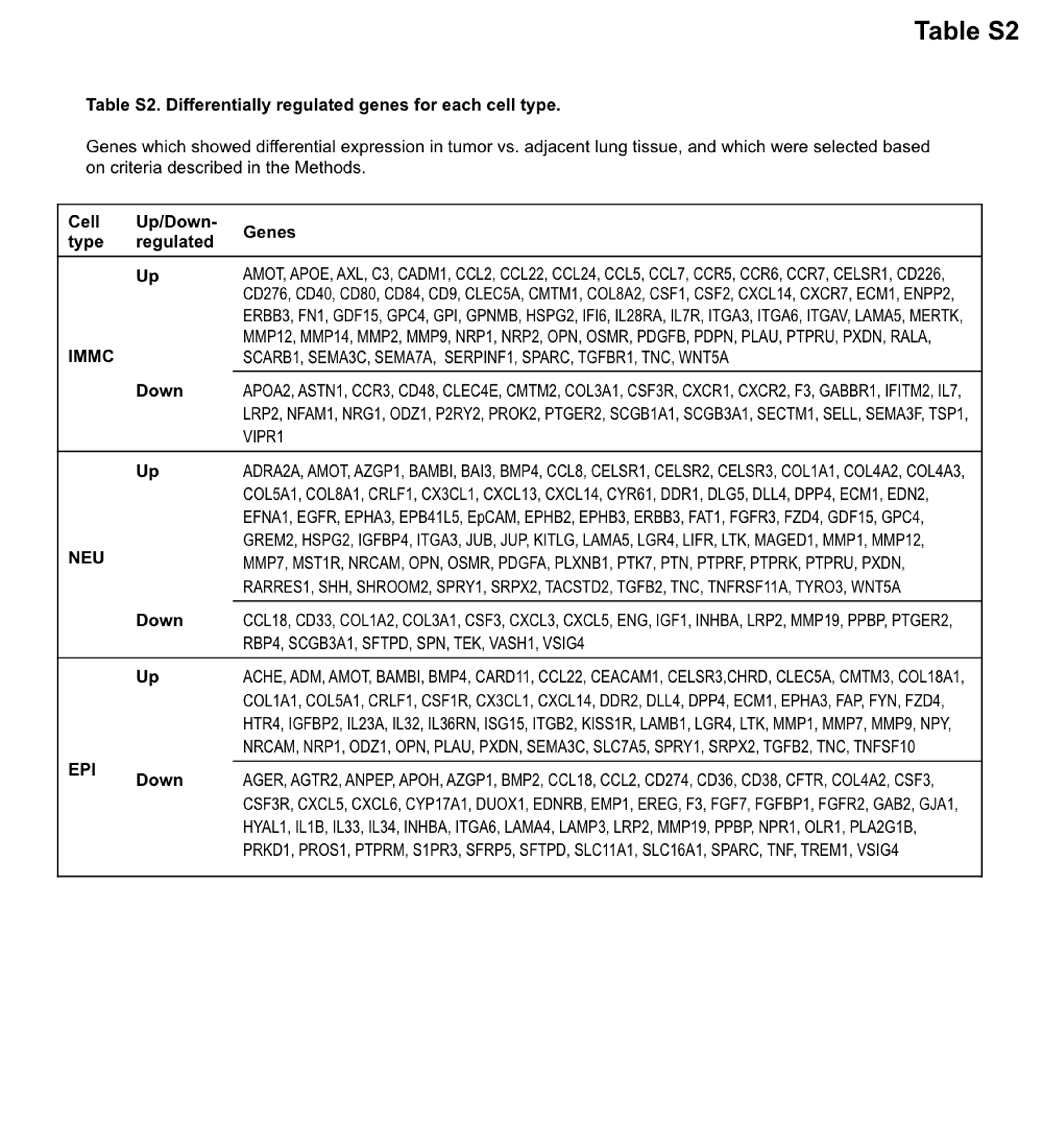

Supplement: S2 Table — (TIF) [file pone.0129123.s009.tif]
